# PB2 mutations arising during H9N2 influenza evolution in the Middle East confer enhanced replication and growth in mammals

**DOI:** 10.1371/journal.ppat.1007919

**Published:** 2019-07-02

**Authors:** Yasuha Arai, Norihito Kawashita, Madiha Salah Ibrahim, Emad Mohamed Elgendy, Tomo Daidoji, Takao Ono, Tatsuya Takagi, Takaaki Nakaya, Kazuhiko Matsumoto, Yohei Watanabe

**Affiliations:** 1 Department of Infectious Diseases, Graduate School of Medical Sciences, Kyoto Prefectural University of Medicine, Kyoto, Japan; 2 Faculty of Science and Engineering, Kindai University, Osaka, Japan; 3 Graduate School of Pharmaceutical Sciences, Osaka University, Osaka, Japan; 4 Department of Microbiology and Immunology, Faculty of Veterinary Medicine, Damanhour University, Damanhour, Egypt; 5 The Institute of Scientific and Industrial Research, Osaka University, Osaka, Japan; University of Michigan, UNITED STATES

## Abstract

Avian influenza virus H9N2 has been endemic in birds in the Middle East, in particular in Egypt with multiple cases of human infections since 1998. Despite concerns about the pandemic threat posed by H9N2, little is known about the biological properties of H9N2 in this epicentre of infection. Here, we investigated the evolutionary dynamics of H9N2 in the Middle East and identified phylogeny-associated PB2 mutations that acted cooperatively to increase H9N2 replication/transcription in human cells. The accumulation of PB2 mutations also correlated with an increase in H9N2 virus growth in the upper and lower airways of mice and in virulence. These mutations clustered on a solvent-exposed region in the PB2-627 domain in proximity to potential interfaces with host factors. These PB2 mutations have been found at high prevalence during evolution of H9N2 in the field, indicating that they have provided a selective advantage for viral adaptation to infect poultry. Therefore, continuous prevalence of H9N2 virus in the Middle East has generated a far more fit or optimized replication phenotype, leading to an expanded viral host range, including to mammals, which may pose public health risks beyond the current outbreaks.

## Author summary

The G1-like clade of H9N2 influenza viruses can undergo genetic reassortment with other influenza virus subtypes to produce novel zoonotic viruses, such as the Gs/GD lineage H5N1, H7N9, H10N8, and H5N8 viruses. Since 1998, the G1-like subclade of H9N2 influenza virus has been widely circulating in birds in Central Asia and the Middle East and a number of human cases have been reported. However, little is known about the biological properties of H9N2 viruses in this epicentre of infection. Our data showed that, during about two decades of evolution in nature, G1-like subclade strains evolved to produce strains with appreciably higher replication phenotypes in Central Asia and the Middle East, which led to their expanded host range, including to humans. Therefore, G1-like subclade strains in these areas may accumulate mutations to produce novel viruses and the large gene pool in these areas would enable reassortment with other influenza viruses. This study indicated the need for studies of H9N2 viruses in such areas to monitor their evolutionary dynamics and possible genetic changes.

## Introduction

The avian influenza (AI) virus H9N2 subtype was first identified in South China in 1994 [1]. H9N2 viruses have now spread worldwide and, since 1997, have been prevalent in poultry along with the H5N1 virus. H9N2 infections cause a decline in egg production, with occasional high mortality in poultry [2]. Human H9N2 infections have been reported repeatedly, posing a serious threat to public health [2, 3]. The internal genes of H9N2 viruses are frequently packaged in zoonotic virus reassortants produced from co-infections by H9N2 and other subtype AI viruses (i.e., H5N1 Gs/GD lineage, H7N9, H10N8, H5N8 and H5N6 viruses) [1, 4–6]. This production of various subtype AI viruses containing H9N2 internal genes with their accumulated mutations raises a concern that H9N2 virus may generate novel reassortants and pandemic viruses.

H9N2 virus strains are classified in Eurasian and American lineages. The Eurasian lineage is divided into several clades, with the majority of strains classified in G1-like and Y280-like clades. G1-like clade viruses have mainly circulated in South China, Central Asia and the Middle East and are represented by the prototype A/quail/Hong Kong/G1/1997 virus. The G1-like clade in Central Asia and the Middle East has diversified further into four co-circulating subclades (designated A-D), with occasional intra-subclade reassortments [7]. The Y280-like clade viruses have spread throughout China and are represented by the prototype A/duck/Hong Kong/Y280/1997 virus.

It has been reported that the G1-like subclades were introduced into Central Asia and the Middle East from East Asia after 1998 [7] and spread among poultry in a number of countries, including Pakistan, Afghanistan, Iran, Iraq, Saudi Arabia, UAE, Israel, Jordan and Egypt [7–11]. G1-like subclades A and B (G1-A and G1-B) have predominated in birds in these areas and a unique G1-A/B reassortant emerged after 2006 [7]. The G1-A/B reassortant was first identified in Egypt in 2010 and swiftly became endemic in domestic poultry there [12, 13]. Thus far, four cases of human H9N2 infections have been reported in Egypt [2]. The H5N1 virus has also circulated in Egypt in a wide range of birds [13] and caused 67% of the recent human H5N1 infection cases worldwide since 2009 (308 cases, as of 21 September 2018, according to WHO). Egypt is now an epicenter for the generation of novel H9N2 and H5N1 viruses and possible reassortments between these two viruses.

The evolutionary dynamics of the H9N2 and H5N1 viruses that co-circulate in the Middle East, centered on Egypt, needs to be understood to facilitate strategies for the control of these viruses. Several studies have reported on the infectivity and transmissibility of the Middle Eastern H5N1 viruses [14, 15] and characterized their human-adaptation signatures [16, 17]. In contrast, thus far, most studies of H9N2 viruses in the area have been limited to genetic analyses [13, 18, 19]. The biological properties of these viruses remain largely unknown.

We previously reported that the H9N2 G1-A/B reassortant isolated in Egypt replicates to markedly high titers in human cells and in mice [20], indicating that the H9N2 virus may expand its host range to mammals. In this study, we investigated all phylogeny associated-mutations that have accumulated during H9N2 evolution in the Middle East and/or Central Asia, and the effect of these mutations on the biological properties of the H9N2 virus, including its adaptation to infect humans.

## Results

### The H9N2 G1-A/B reassortant PB2 gene contributed to higher polymerase activity in human cells than other zoonotic influenza viruses

In our previous study, we found that a representative strain of the G1-A/B reassortant, A/chicken/Egypt/CL42/2013 (H9N2) (referred to here as EG/2013), replicated at a high level in human cells and in mice. EG/2013 replication was higher than that of a H5N1 clade 2.2.1 strain carrying the representative human adaptation PB2-E627K mutation [20]. This implied that the G1-A/B reassortant may have acquired greater fitness to infect mammalian species, including humans, than other AI viruses.

To elucidate the replication capacity of the G1-A/B reassortant in human cells, the polymerase activity of the EG/2013 (H9N2) virus in human 293T cells was compared to that of three other zoonotic AI viruses; i.e., A/quail/Hong Kong/G1/1997 (H9N2), A/duck/Egypt/D1Br/2007 (H5N1) and A/Shanghai/1/2013 (H7N9). A/quail/Hong Kong/G1/1997 (G1/1997) (H9N2) is the prototype virus of the G1-like clade. A/duck/Egypt/D1Br/2007 (EG/2007) (H5N1) is an ancestral strain of clade 2.2.1 that is unique to Egypt: most clade 2.2.1 field isolates, including EG/2007, have the PB2-E627K mutation [21]. A/Shanghai/1/2013 (Sh/2013) (H7N9) is an ancestral strain of a novel H7N9 virus that has six internal genes derived from an H9N2 virus: some human H7N9 isolates, including Sh/2013, have acquired the PB2-E627K mutation [22].

**Fig 1:**
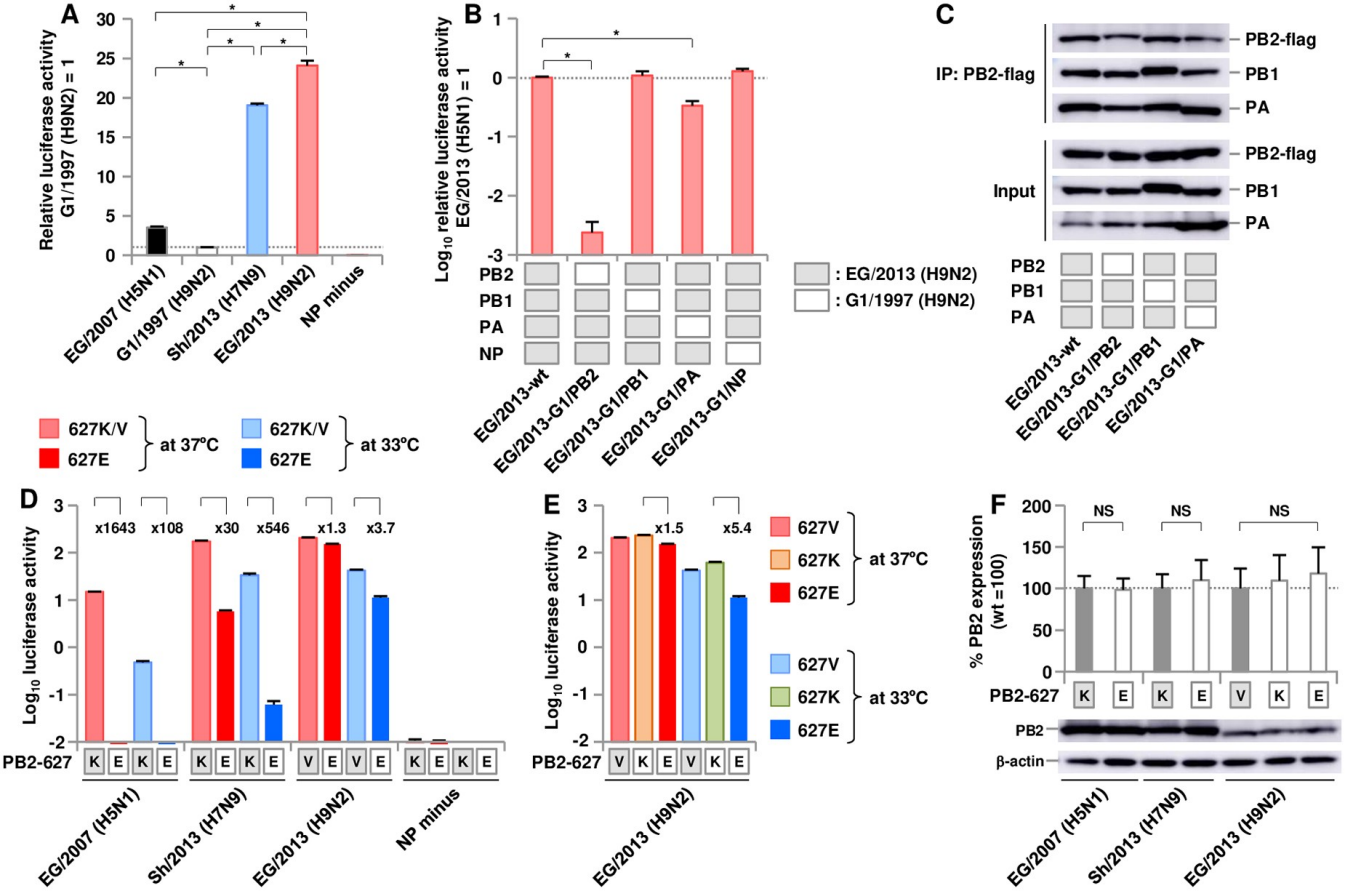
Polymerase activity of zoonotic avian influenza viruses in human cells. Viral polymerase activity was measured by minigenome assays in human 293T cells. (A) Polymerase activity of zoonotic avian influenza viruses at 33°C. The data are expressed relative to the results for G1/1997 (H9N2). (B) Polymerase activity at 33°C of reassorted polymerase complexes composed of one subunit from EG/2013 (H9N2) and the other subunits from ancestral G1/1997 (H9N2). The data are expressed relative to the results for EG/2013-wt. (C) Western blot analysis of reassortant polymerase complexes shown in B without (Input) and with (IP: PB2-flag) immunoprecipitation with PB2-flag. Representative images are shown. (D) Polymerase activity of PB2-K627E revertants from EG/2007 (H5N1) and Sh/2013 (H7N9), and of the PB2-V627E revertant from EG/2013 (H9N2) at 37°C and 33°C. (E) Polymerase activity of the PB2-V627K mutant from EG/2013 (H9N2) at 37°C and 33°C. The values of the fold reduction relative to wild-type (D) and to the PB2-V627K mutant (E) are shown. (F) Quantification of PB2 expression levels in 293T cells. The cells were transfected with plasmids expressing the indicated non-tagged PB2, and cell lysates were analyzed by western blotting using anti-PB2 antibody. Representative images are shown. Each data point is the mean ± SD of three independent experiments. Asterisks indicate a P value < 0.01. NS indicates no statistically significant difference.

Minigenome assays showed that the EG/2013 (H9N2) polymerase complex (PB2, PB1, PA and NP) had significantly higher polymerase activity in 293T cells than that of the three other AI viruses in this study (Fig 1A). To determine which polymerase subunits contribute to the high polymerase activity of EG/2013, the polymerase activity of polymerase complexes composed of different combinations of the polymerase subunits of EG/2013 and ancestral G1/1997 were examined. A polymerase complex with G1/1997 PB2 in the EG/2013 background had a significant decrease in polymerase activity by > 2 logs (Fig 1B), whereas a polymerase complex with G1/1997 PA had only a small reduction in polymerase activity. In contrast, G1/1997 PB1 and NP had little effect on EG/2013 polymerase activity. Also, polymerase complexes with PB2-flag were precipitated from the transfected cell lysates and the amount of PB1 and PA co-immunoprecipitated with PB2 was analyzed by western blotting (Fig 1C). Similar amounts of PB1 and PA in these polymerase complexes were co-immunoprecipitated with PB2, verifying that reassortment did not affect trimeric complex formation. These results indicated that EG/2013 PB2 was a major determinant of the high EG/2013 polymerase activity in human cells.

G1-A/B reassortants, including EG/2013, generally carry the PB2-E627V mutation [13], which has been recently identified as a mammalian-adaptation mutation in H5N1 clade 2.3.4 viruses [23]. However, this mutation has not been studied in the H9N2 background. The effect of the PB2-V627E reverse mutation on EG/2013 polymerase activity was investigated by minigenome assays at 37°C and 33°C, which are temperatures characteristic of the human lower and upper respiratory tract, respectively. For comparison, we investigated the effect of the PB2-K627E reverse mutation on the polymerase activity of EG/2007 (H5N1) and Sh/2013 (H7N9). The PB2-K627E reverse mutation decreased EG/2007 (H5N1) polymerase activity by > 2 logs at 37°C and 33°C (Fig 1D). A similar effect was observed in the Sh/2013 (H7N9) background, with a l-3 log reduction. In contrast, EG/2013 (H9N2) polymerase activity was barely affected by the PB2-V627E reverse mutation, with only 1.3-fold and 3.7-fold reductions at 37°C and 33°C, respectively.

To investigate the different amount of the effects mediated by V627E and K627E, the effect of K627E was investigated in the EG/2013 background. As with V627E, K627E caused only 1.5-fold and 5.4-fold reductions in polymerase activity (Fig 1E), indicating that the effects of K627E and V627E on EG/2013 polymerase activity were similar. These results indicated that the levels of EG/2007 (H5N1) and Sh/2013 (H7N9) polymerase activity were mediated by E627K, but the higher polymerase activity of EG/2013 (H9N2) was not fully dependent on E627V. Western blotting analysis confirmed similar expression of wild-type PB2 and the K/V627E mutants (Fig 1F).

However, the apparently distinct antigenicity of the different virus subtypes prevented our direct comparison of the PB2 expression levels of EG/2007 (H5N1), sh/2013 (H7N9) and EG/2013 (H9N2). Therefore, the expression of PB2-flag, which was not affected by PB2 antigenic properties was analyzed further. The PB2-flag expression levels of EG/2007 (H5N1) and EG/2013 (H9N2) were similar, although they were higher than sh/2013 (H7N9) (S1 Fig). Wild-type and mutant PB2-flag were also expressed at similar levels. Comparable PB2-flag expression was also found between G1/1997 (H9N2) and EG/2013 (H9N2) (Fig 1C, input). These data indicated that the K/V627E substitutions did not affect PB2 expression levels and that the higher polymerase activity of EG/2013 (H9N2) was not due to a higher PB2 expression level than in the other AI viruses. Taken together, these results suggested that other PB2 mutations, that had accumulated during the evolution of H9N2 G1-A/B, may contribute to EG/2013 polymerase activity in human cells.

### The G1-A/B reassortant has accumulated multiple PB2 mutations during its evolution in the Middle East

**Fig 2:**
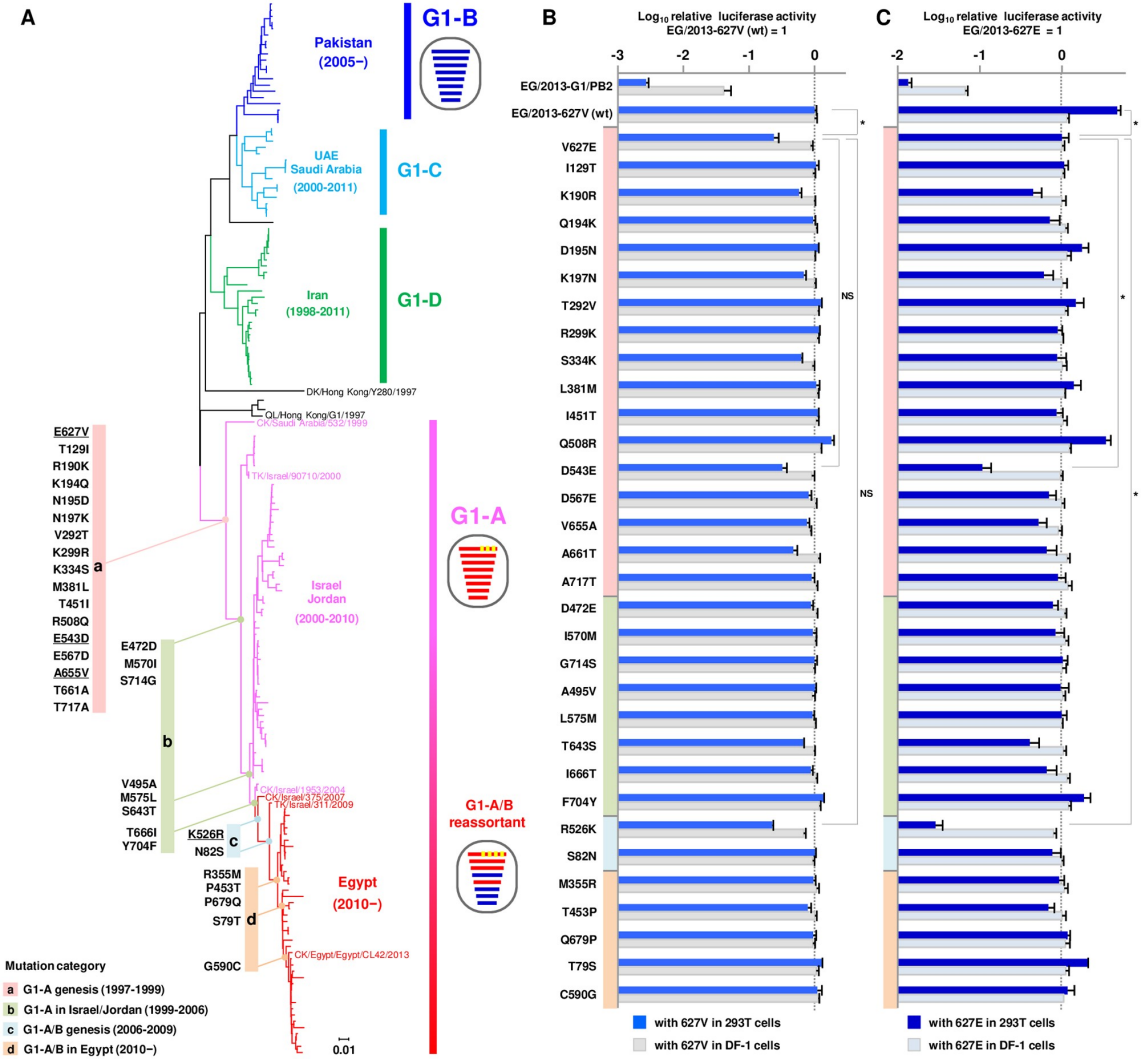
Phylogeny of the PB2 gene in H9N2 G1-like subclades isolated in the Middle East and Central Asia. (A) Phylogenetic tree of the PB2 genes of H9N2 G1-like subclades isolated in the Middle East and Central Asia, including the G1-A/B reassortant. The tree was reconstructed from nucleotide sequences of the PB2 genes of the Middle Eastern and Central Asian reference strains and of the prototype G1/1997, EG/2013 and A/duck/Hong Kong/Y280/1997 (i.e., the prototype Y280-like clade). CK, DK, QL and TK in the virus strain names denote chicken, duck, quail and turkey hosts. Phylogeny-associated PB2 mutations acquired during the evolution of G1-A/B are shown beside each branch, with the mutations grouped in the four time periods during which they were identified (designated mutation categories a-d). The four mutations that were shown in this study to act cooperatively in expansion of the host range to humans are underlined. Virus particles are represented by ovals, with horizontal bars representing the eight gene segments (from top to bottom: PB2, PB1, PA, HA, NP, NA, M and NS). Segments in the descendent viruses are colored according to the corresponding source viruses to illustrate the gene ancestry through reassortment. Yellow in segment 1 (PB2) indicates the four phylogeny-associated mutations that contribute to the expanded viral host range to humans. (B and C) Polymerase activities of EG/2013-wt and the PB2 revertants carrying single mutations in human 293T cells at 33°C and in avian DF-1 cells at 37°C, without the PB2-V627E mutation (B) and with the PB2-V627E mutation (C). The data are expressed relative to the results for EG/2013-627V (wt) (B) and for EG/2013-V627E (C). Asterisks indicate a P value < 0.01. NS indicates no statistically significant difference. Colors of the vertical bars indicate the four groups of strains based on when the mutations were identified during the evolution of G1-A/B, as shown in A.

To trace evolution of the PB2 gene in the G1-A/B reassortant, we analyzed the phylogeny of PB2 genes, including the PB2 genes of the G1-like prototype A/quail/Hong Kong/G1/1997 and the descendant G1-like subclades that were isolated in Central Asia and the Middle East from 1998 to 2015, including EG/2013. The PB2 genes in viruses isolated in Central Asia and the Middle East clustered in several groups (Fig 2A). These largely corresponded to the HA monophyletic groups (HA gene subclades A-D) (S2 Fig). The topology of the PB2 and HA gene phylogenetic trees indicated a major intra-subclade reassortment between G1-A and G1-B viruses. It has been reported that the G1-A/B reassortant emerged in Israel and Jordan, with the PB2, PB1, PA and NP genes from G1-A and the other genes from G1-B, and has become dominant there since 2006 [7]. Egyptian H9N2 viruses descended from the Israel/Jordan virus group and form a regionally distinct subgroup with a few early Israeli strains isolated since 2010 (Fig 2A), indicating that the Egyptian H9N2 virus originated from the G1-A/B reassortant that was prevalent in Israel and Jordan. The G1-like subclades in Central Asia and the Middle East were mostly isolated from chickens, but also from birds, including turkeys, quails, ducks, ostriches, pheasants and pigeons. The isolates from each bird species were interspersed throughout the H9N2 phylogenetic tree without distinct clustering, indicating a wide dispersion of these viruses in birds in these geographic areas.

We then searched for the PB2 mutations that the G1-A/B reassortant had accumulated during its evolution in the field, with reference to the ancestral G1/1997 virus. A total of 32 amino acid substitutions were identified and grouped in four mutation categories, designated a-d (Fig 2A). Category a contained 17 substitutions, including E627V, that had all been detected in the oldest H9N2 G1-A strain in the Middle East (A/chicken/Saudi Arabia/532/1999) and all its descendants. Category b contained strains with 8 substitutions that were identified during the G1-A prevalence in Israel and Jordan during 2000–2006. Category c contained 2 substitutions that were identified in G1-A/B reassortants in 2006–2009. Category d contained 5 substitutions that were maintained after incursion of the G1-A/B reassortant into Egypt around 2010. The G1-A/B reassortant that has predominated in Egypt since then and almost all its descendants have retained these substitutions, indicating that they may have contributed to the establishment of the G1-A/B reassortant in poultry.

### Phylogeny-associated PB2 mutations increased the polymerase activity of the G1-A/B reassortant in human cells

To determine the effect of each mutation on viral polymerase activity, we introduced a reverse mutation of each mutation that had been identified into the EG/2013 background, both with and without PB2-V627E, and measured the polymerase activities of the revertants by minigenome assays in avian DF-1 cells at 37°C and in 293T cells at 33°C. As a reference, a reassorted polymerase complex composed of PB2 from G1/1997 and PB1, PA and NP from EG/2013 (referred to here as EG/2013-G1/PB2) was included in these assays. In avian cells at 37°C, none of the single reverse mutations had a significant effect on EG/2013 polymerase activity (Fig 2B and 2C). In contrast, in human cells at 33°C, the D543E and R526K reverse mutations decreased the EG/2013 polymerase activity to levels comparable to that of the V627E reverse mutation (Fig 2B). The effect was greater when the mutation was combined with V627E (Fig 2C). The K190R and T643S reverse mutations only showed moderate effects when combined with V627E. The polymerase activity of these single revertants was higher than that of the reference EG/2013-G1/PB2, indicating that multiple mutations contributed to the high polymerase activity of the G1-A/B reassortant. At 37°C, the effects were negligible, except that the R526K reverse mutation showed a noticeable effect in reducing EG/2013 polymerase activity in human cells (S3 Fig). These PB2 mutants had equivalent expression levels, although there were some small statistically insignificant changes in these levels (S4 Fig). There was no apparent difference in PB2 expression between the wild-type and the mutants with appreciably reduced polymerase activity (i.e., V627E, D543E, R526K, K190R and T643S), indicating that their lower polymerase activity was not due to a decrease in PB2 expression. Together, these results suggested that the reverse mutations of the phylogeny-associated PB2 mutations in this study, singly and together with V627E, decreased EG/2013 polymerase activity in human cells to a greater degree at the lower 33°C temperature.

### PB2-E627V/E543D/A655V/K526R mutations acted cooperatively to produce the high polymerase activity of the G1-A/B reassortant in human cells

**Fig 3:**
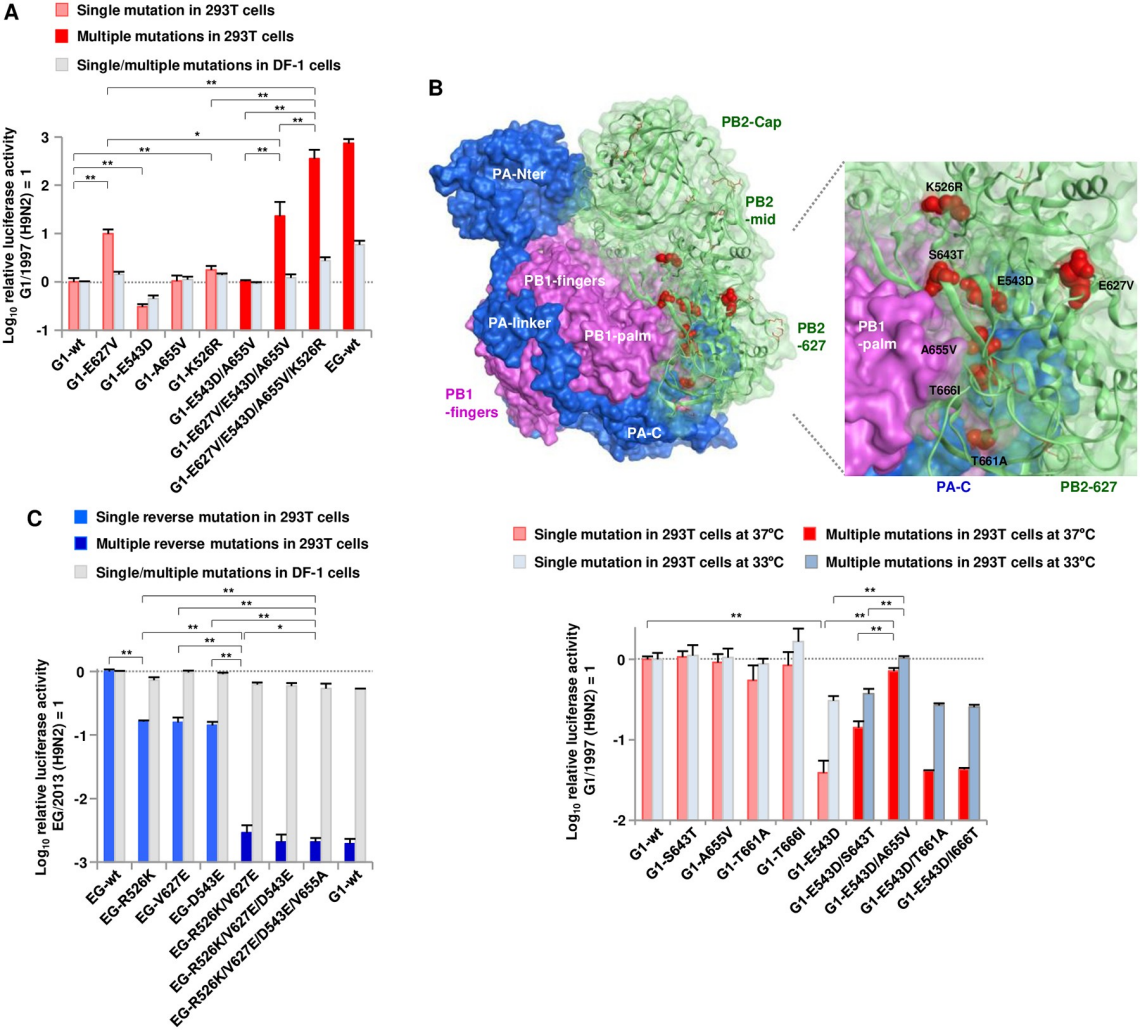
Cooperative effects of mutations on viral polymerase activity in avian and human cells. Viral polymerase activity was measured by minigenome assays in avian DF-1 cells and human 293T cells. (A and C) Polymerase activity in ancestral G1/1997 (A) and EG/2013 backgrounds (C). Polymerase complexes carrying the indicated PB2 mutations, singly or in combination, were assayed in 293T cells at 33°C and in DF-1 cells at 37°C. The data are expressed relative to the results for G1/1997-wt (A) and EG/2013-wt (C). (B) (Upper panel) Transparent surface diagram of the G1/1997 polymerase complex color-coded showing PB2 (green), PB1 (pink), PA (blue) and the mutations identified in this study (red), including the putative complementary PB2 mutations located in the vicinity of the PB2-543 residue. (Lower panel) Polymerase activity of G1/1997 polymerase complexes carrying the putative complementary PB2 mutations, singly or coupled with E543D, in 293T cells at 37°C and at 33°C. The data are expressed relative to the results for G1/1997-wt. Each data point is the mean ± SD of three independent experiments. Double and single asterisks indicate a P value < 0.01 and < 0.05, respectively.

Based on the effects of the reverse mutations in the EG/2013 background, we selected three PB2 mutations (i.e., E627V, E543D and K526R) for further study and introduced them as forward mutations into the ancestral G1/1997 background to assess their effects on polymerase activity. The E627V and K526R mutations significantly increased G1/1997 polymerase activity in human cells (Fig 3A). But, the PB2-E543D mutation unexpectedly reduced the polymerase activity in human and avian cells, implying that PB2-E543D needed a compensatory mutation(s) that acted cooperatively in the G1/1997 background. Therefore, we modeled the G1/1997 polymerase complex structure (PDB: 4WSB as the modeling template) and searched for PB2 mutations that were structurally close to or related to the PB2-543 residue. Four mutations (i.e., S643T, A655V, T661A and T666I) were identified as putative compensatory mutations (Fig 3B, upper panel) and their cooperative effects with PB2-E543D were examined. As single mutations, all four had little effect on G1/1997 polymerase activity (Fig 3B, lower panel): only A655V completely reversed the reduction caused by E543D. The E543D/A655V double mutation restored G1/1997 polymerase activity to a level comparable to G1/1997-wt, indicating that these mutations acted cooperatively on G1/1997 polymerase activity.

In subsequent experiments, we introduced the E543D forward mutation together with the A655V compensatory mutation into the G1/1997 background. In addition, the D543E reverse mutation was introduced into the EG/2013 background, either singly or together with V655A: EG/2013 carried PB2-655V and, therefore, D543E did not necessarily require V655A in this background. In addition, the phylogenetically oldest G1-A strain (A/chicken/Saudi Arabia/532/1999) carried E627V, E543D and A655V (Fig 2A), which prevented us analyzing the phylogenetic order of these tree mutations. Therefore, multiple forward mutations were combined in their putative phylogenetic order (i.e., E627V, E543D/A655V and K526R) and multiple reverse mutations were combined in their putative phylogenetic order [i.e., R526K, V627E and D543E(/V655A)].

The cooperative effects of E627V, E543D/A655V and K526R on polymerase activity were then investigated. These mutations acted synergistically to increase G1/1997 polymerase activity by > 2 logs in human cells (Fig 3A): the E627V/E543D/A655V/K526R quadruple mutation had the highest polymerase activity, at a level comparable to that of EG/2013-wt. The relative polymerase activities were G1/1997-wt < E627V < E627V/E543D/A655V < E627V/E543D/A655V/K526R = EG/2013-wt. Conversely, the reverse mutations acted cooperatively to decrease EG/2013 polymerase activity by > 2 logs in human cells (Fig 3C). The R526K/V627E/D543E/V655A mutation had the lowest polymerase activity; i.e., at an undetectable level in minigenome assays that was comparable to that of G1/1997-wt. The relative polymerase activities were EG/2013-wt > R526K = V627E and D543E > R526K/V627E > R526K/V627E/D543E/V655A = G1/1997-wt. The > 2 log difference in polymerase activity between the wild-type and quadruple mutant corresponded to that between EG/2013 and EG/2013-G1/PB2 (Fig 1B), indicating that the increase in EG/2013 polymerase activity was mostly due to the quadruple mutation. In contrast, in avian cells, the quadruple mutation showed an appreciably lower effect in both the G1/1997 and EG/2013 backgrounds. These results suggested that E627V, E543D/A655V and K526R acted cooperatively to produce the high polymerase activity of the G1-A/B reassortant, which was more prominent in human cells than in avian cells.

**Fig 4:**
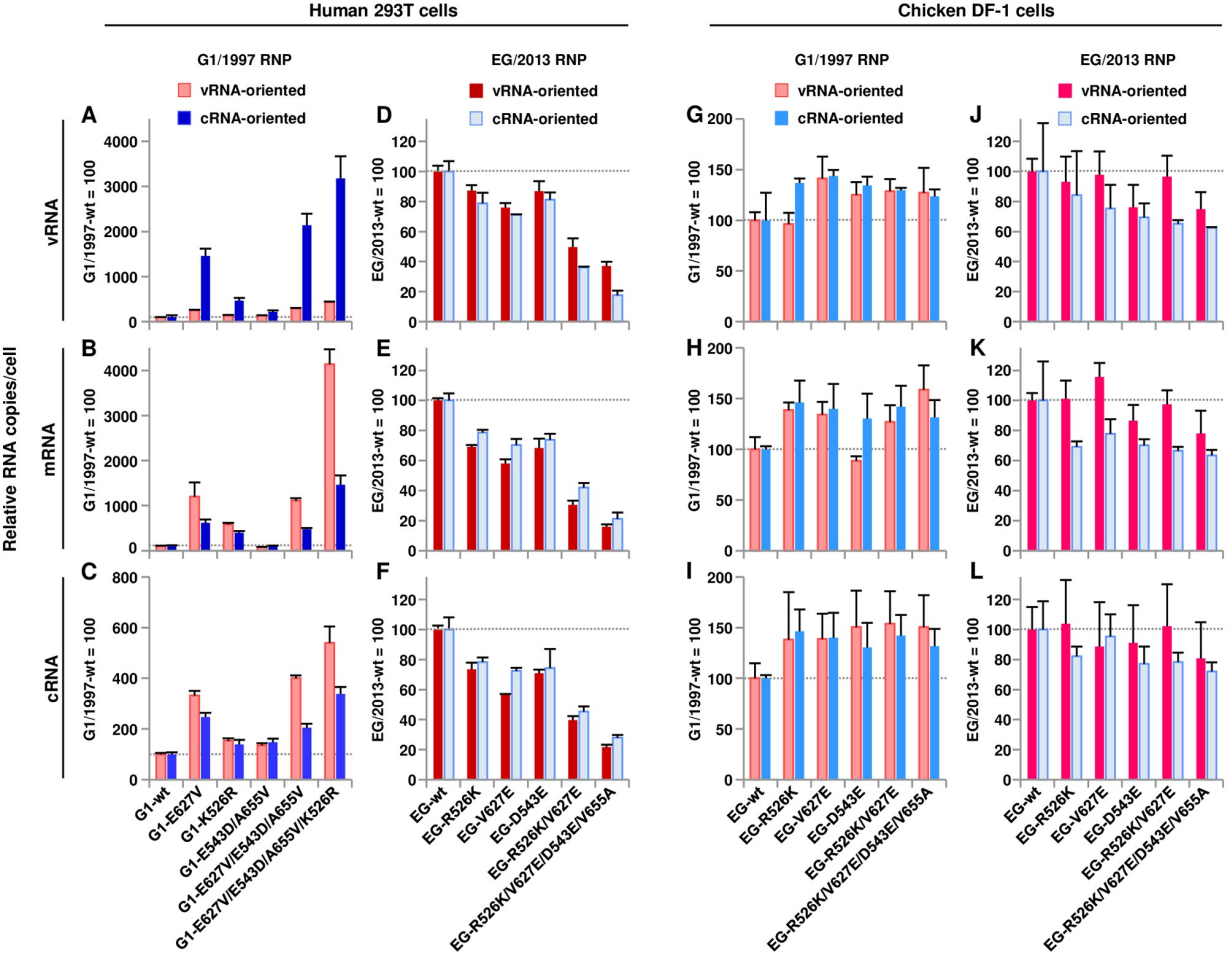
Effect of PB2 mutations on RNA synthesis from RNPs reconstituted in human and avian cells. Viral RNA synthesis was assayed by vRNA-oriented and cRNA-oriented NA RNP reconstitution assays in 293T cells at 33°C (A-F) and in DF-1 cells at 37°C (G-L). At 24 h post-transfection, the viral RNAs were extracted and the levels of vRNA (A, D, G and J), mRNA (B, E, H and K) and cRNA (C, F, I and L) were quantified by strand-specific real-time RT-PCR. (A-C and G-I) Viral RNA levels from G1/1997 RNPs with PB2 forward mutations. The data are expressed relative to the results for G1/1997-wt. (D-F and J-L) Viral RNA levels from EG/2013 RNPs with PB2 reverse mutations. The data are expressed relative to the results for EG/2013-wt. Each data point is the mean ± SD of three independent experiments.

The levels of viral vRNA, cRNA and mRNA syntheses were assayed by vRNA-oriented and cRNA-oriented RNP reconstitution assays in 293T and DF-1 cells. mRNA can be transcribed directly from vRNA in vRNA-oriented RNP reconstitution assays, whereas cRNA must be replicated to vRNA before it can be transcribed to mRNA in the cRNA-oriented RNP reconstitution assays. Therefore, cRNA-oriented RNP reconstitution assays enabled us to directly analyze the effect of polymerase-mediated replication activity [16, 24]. Strand-specific qRT-PCR showed that, in human cells, G1/1997 RNPs reconstituted with the PB2 forward mutants produced higher levels of vRNA, cRNA and mRNA than wild-type, both in vRNA-oriented and cRNA-oriented RNP reconstitution assays (Fig 4A–4C). However, EG/2013 RNPs reconstituted with PB2 reverse mutants produced lower levels of vRNA, cRNA and mRNA than wild-type in vRNA-oriented and cRNA-oriented RNP reconstitution assays (Fig 4D–4F). These mutations had more dramatic effects on vRNA synthesis from cRNA templates than on cRNA synthesis from vRNA templates in the G1/1997 genetic background (Fig 4A vs 4C), despite the less dramatic effects observed in the EG/2013 genetic background. In contrast, the mutations had appreciably less effect on viral RNA synthesis in avian cells (Fig 4G–4L). The strand specificity of the qRT-PCR primers was confirmed using *in vitro* transcribed RNA templates (S5 Fig): the vRNA primer set detected only <0.01% of both cRNA and mRNA, the cRNA primer set also detected only <0.01% of vRNA and mRNA, and the mRNA primer set detected only <0.01% of vRNA and minimally detected 1.5% of cRNA. In total, these results suggested that the E627V, K526R and E543D/A655V mutations acted cooperatively to increase both transcription and replication activity in the G1-A/B reassortant. This effect was more prominent in human cells than in avian cells.

### The PB2-E627V/E543D/A655V/K526R mutations all contributed to efficient replication of the G1-A/B reassortant in human cells

**Fig 5:**
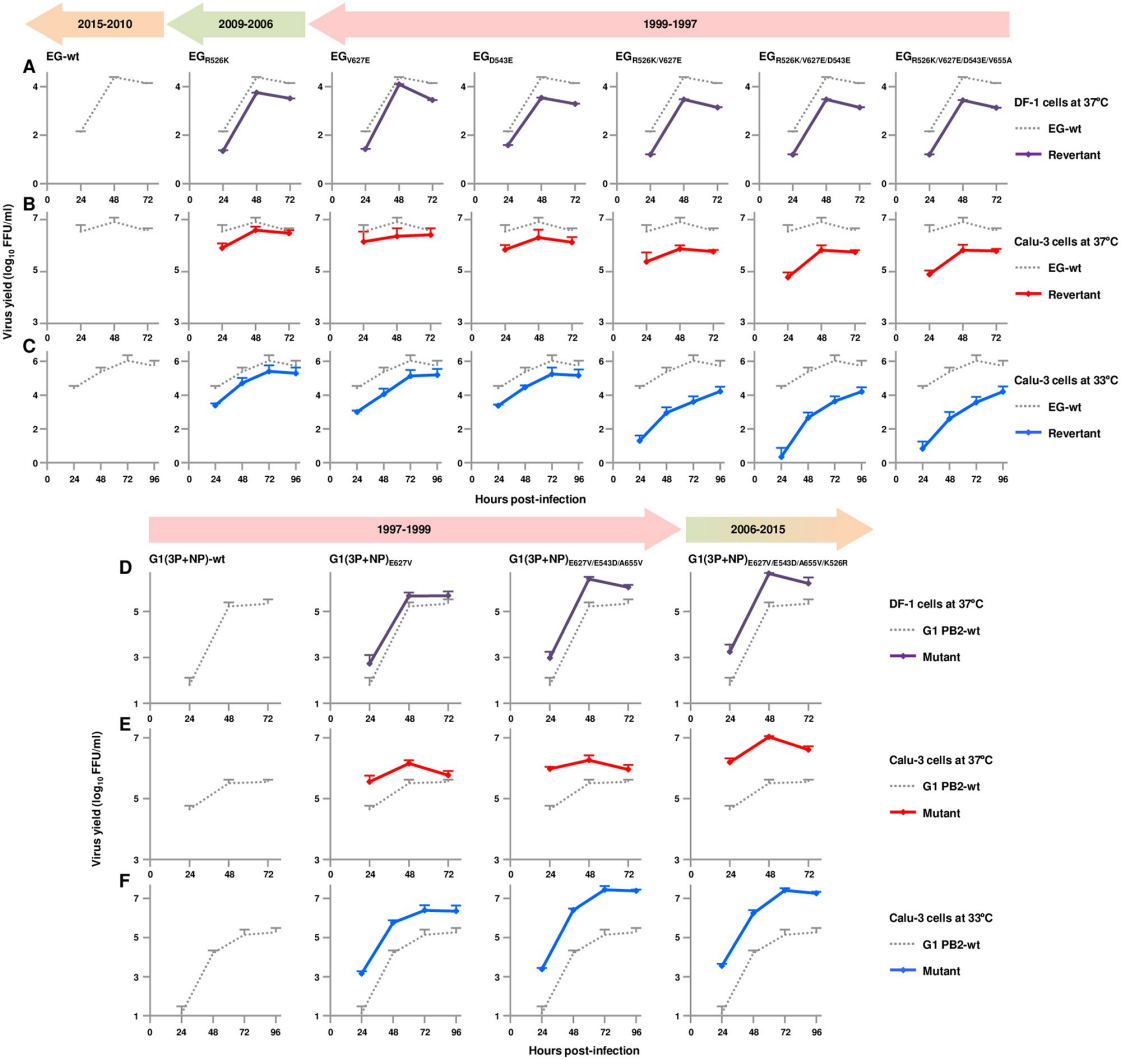
Replication kinetics of EG/2013 and G1/1997 viruses carrying PB2 mutations in avian and human cells. (A-C) Avian DF-1 cells (A) and human Calu-3 cells (B and C) were infected with EG/2013 virus or the indicated PB2 revertants at an MOI of 0.005 and 0.001, respectively, and incubated at 37°C (A and B) or 33°C (C). (D-F) DF-1 cells (D) and Calu-3 cells (E and F) were infected with reassorted G1(3P+NP) virus carrying PB2 (wild-type or with the indicated mutations), PB1, PA and NP from G1/1997, with the other genes from EG/2013, at an MOI of 0.005 and 0.001, respectively, and incubated at 37°C (D and E) or 33°C (F). Virus titers at the indicated times post-infection were determined by FFU assays. Each data point is the mean ± SD of three independent experiments. Colors of the arrows above the figures indicate the time periods when the mutations were identified during the evolution of G1-A/B, as shown in Fig 2A.

We investigated the effect of the amino acids at PB2 residues 627, 543, 655 and 526 on the replication kinetics of the G1-A/B reassortant using recombinant viruses in the EG/2013 background. DF-1 cells and human airway epithelial Calu-3 cells were infected with EG/2013-wt and the revertants and the replication kinetics of the viruses were compared. In both cell types, the revertants produced significantly lower progeny virus titers than EG/2013-wt (Fig 5A–5C), with consistently greater effects in Calu-3 cells at 33°C (Fig 5C). The phylogenetically earliest revertant, R526K/V627E/D543E(/V655A), designated EG_R526K/V627E/E543D(/V655A)_, had the lowest progeny virus yield in both cell types by > 3 logs in human cells at early times post-infection: EG/2013-wt > R526K = V627E and D543E > R526K/V627E > R526K/V627E/D543E(/V655A).

The role of the PB2 amino acid at residues 627, 543, 655 and 526 in the G1/1997 background on virus replication was studied next. For these experiments, we used a reassortant virus carrying polymerase-associated genes (PB2, PB1, PA and NP) from G1/1997 and the other genes from EG/2013 to assess polymerase-specific effects on virus replication. The reassortant virus was designated G1(3P+NP). The G1(3P+NP) mutants produced significantly higher virus titers than G1(3P+NP)-wt in both DF-1 cells and Calu-3 cells (Fig 5D–5F), with more significant effects in Calu-3 cells at 33°C (Fig 5F). As in the minigenome assays (Fig 3A), E543D needed to be coupled with the A655V compensatory mutation to act cooperatively to produce increased G1(3P+NP) growth in infected cells (S6 Fig). The most phylogenetically recent mutant genotype, G1(3P+NP)_E627V/E543D/A655V/K526R_, had the greatest progeny virus yield in both cell types: G1(3P+NP)-wt < E627V < E627V/E543D/A655V < E627V/E543D/A655V/K526R. Together, these results suggested that, during about two decades of evolution in the Middle East, the ability of G1-like clade viruses to replicate in avian and human cells gradually increased and the E627V/E543D/A655V/K526R quadruple mutations acted cooperatively in evolution of the G1-A/B reassortant, with the most fit phenotype able to replicate more successfully in human cells at the lower temperature.

### The G1-A/B reassortant carrying PB2-E627V/E543D/A655V/K526R had increased replication and pathogenicity in mice *in vivo*

To assess the relevance of the cooperative effects of the E627V/E543D/A655V/K526R mutation on *in vitro* to *in vivo* infections, BALB/c mice were inoculated intranasally with serial dilutions of two wild-type and four representative mutant strains; i.e., EG/2013-wt, G1(3P+NP)-wt, EG/2013_V627E_, EG/2013_V627E/D543E/V655A/R526K_, G1(3P+NP)_E627V_ and G1(3P+NP)_E627V/E543D/A655V/K526R_. These viruses were categorized into three PB2 groups; i.e., ancestral genotype, G1(3P+NP)-wt and EG/2013_V627E/D543E/V655A/R526K_; intermediate genotype, G1(3P+NP)_E627V_ and EG/2013_V627E_; and current genotype, G1(3P+NP)_E627V/E543D/A655V/K526R_ and EG/2013-wt. The body weight and survival of each infected mouse was monitored.

**Fig 6:**
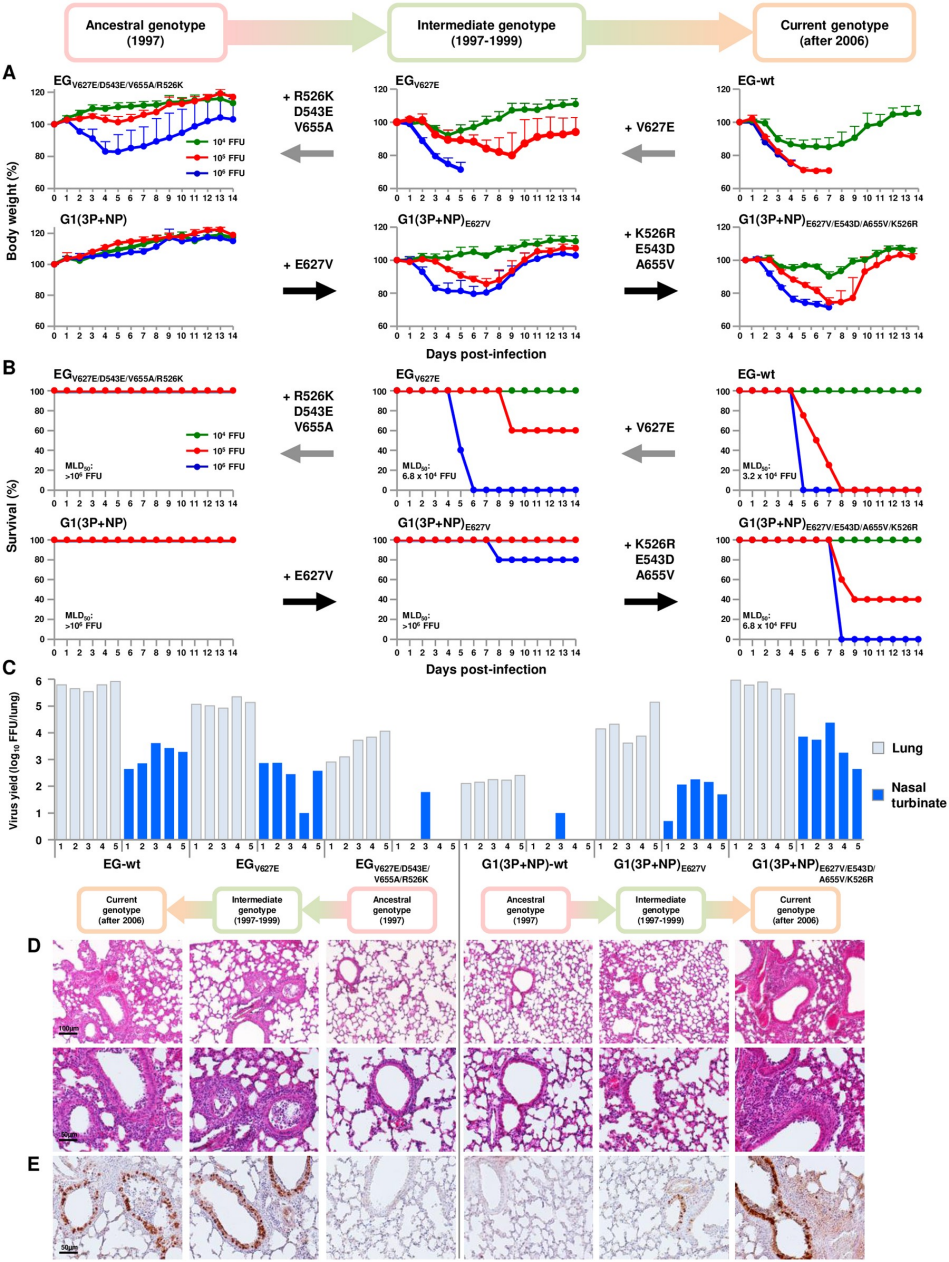
Virulence and replication of EG/2013 and G1/1997 viruses carrying PB2 mutations in the respiratory tract of infected mice. (A and B) Four to five-week-old BALB/c mice (5 mice per group) were inoculated intranasally with 10^4^ to 10^6^ FFU EG/2013 and G1(3P+NP) viruses carrying the indicated PB2 mutations. (A) The body weights of the infected mice were monitored daily for 14 dpi. The mean ± SD of the percent of the initial body weight for each group of mice is shown. (B) Survival of the infected mice. Survival was calculated, including mice that were humanely sacrificed after they had lost more than 30% of their body weight within a few days. The time periods when the genotypes were identified are shown above (A). (C) Viral titers in the lungs and nasal turbinates of mice (5 mice per group) infected with 10^4^ FFU EG/2013 and G1(3P+NP) viruses carrying the PB2 mutations at 3 dpi. Each bar marks the titer in an individual mouse (#1–5). (D, Upper row) Representative low magnification photomicrographs at 3 dpi of hematoxylin and eosin stained sections of lungs from mice infected with the indicated viruses. (D, Lower row) Magnified views of photomicrographs in Upper row. (E) Representative photomicrographs at 3 dpi of immunohistochemically stained sections of lungs from mice infected with the indicated viruses. The time periods when the genotypes were identified are shown above D.

Doses of 10^5^ and 10^6^ FFU of ancestral genotype viruses G1(3P+NP)-wt and EG/2013_V627E/ D543E/V655A/R526K_ caused little body weight change and no mortality (Fig 6A and 6B, left panels), indicating a relatively avirulent phenotype: the 50% mouse lethal dose (MLD_50_) was > 10^6^ FFU for both viruses. In contrast, the same doses of current genotype viruses EG/2013-wt and G1(3P+NP)_E627V/E543D/A655V/K526R_ caused drastic weight loss and 60–100% mortality, resulting in MLD_50_ values of 3.2 x 10^4^ FFU and 6.8 x 10^4^ FFU, respectively (Fig 6A and 6B, right panels). Intermediate genotype viruses EG/2013_V627E_ and G1(3P+NP)_E627V_ had intermediate weight loss and virulence in mice (Fig 6A and 6B, middle panels).

To determine virus titers in the infected mice, groups of five mice were inoculated intranasally with 10^4^ FFU of each virus. Lungs and nasal turbinates were collected 3 d post-infection (dpi) and virus titers in the organs were measured by focus-forming assays. Virus titers in the infected mice were in agreement with their polymerase activity and replication kinetics in human cells *in vitro*. Current genotype EG/2013-wt and G1(3P+NP)_E627V/E543D/A655V/K526R_ viruses replicated most efficiently in the lungs and nasal turbinates (Fig 6C), with titers comparable to each other. In contrast, ancestral genotype viruses G1(3P+NP)-wt and EG/2013_V627E/D543E/V655A/R526K_ replicated with appreciably lower titers in the lungs, and showed clearly attenuated replication in the nasal turbinates: viruses were detected in only one of the five nasal turbinates of the infected mice. EG/2013_V627E_ and G1(3P+NP)_E627V_ viruses showed intermediate replication in the mice.

Pathology studies were carried out on the lungs of infected mice collected at 3 dpi. Ancestral genotype G1(3P+NP) and EG/2013_V627E/D543E/V655A/R526K_ viruses caused a weak inflammatory response in the lungs of the infected mice (Fig 6D). However, current genotype EG/2013-wt and G1(3P+NP)_E627V/E543D/A655V/K526R_ viruses induced notably more severe bronchitis, bronchiolitis and localized bronchointerstitial pneumonia, characterized by occasional foci of bronchial and bronchiolar epithelial cell necrosis, multifocal hemorrhage, and infiltration of inflammatory cells. Intermediate group EG/2013_V627E_ and G1(3P+NP)_E627V_ viruses caused minimal to mild bronchitis or bronchiolitis in infected mice, indicating an intermediate phenotype. The degree of histopathological changes generally corresponded to the amount of H5 antigen detected by immunohistochemistry in the bronchial and bronchiolar areas of the lungs (Fig 6E). Collectively, these results confirmed that the PB2-E627V/E543D/A655V/K526R quadruple mutation produced the most fitness for viral replication in human cells and suggested that the quadruple mutation contributed to an expanded adaptability of the G1-A/B reassortant in mammalian species *in vivo*, even in the upper respiratory tract.

### Effect of mutations on the structure of the G1-A/B polymerase complex

**Fig 7:**
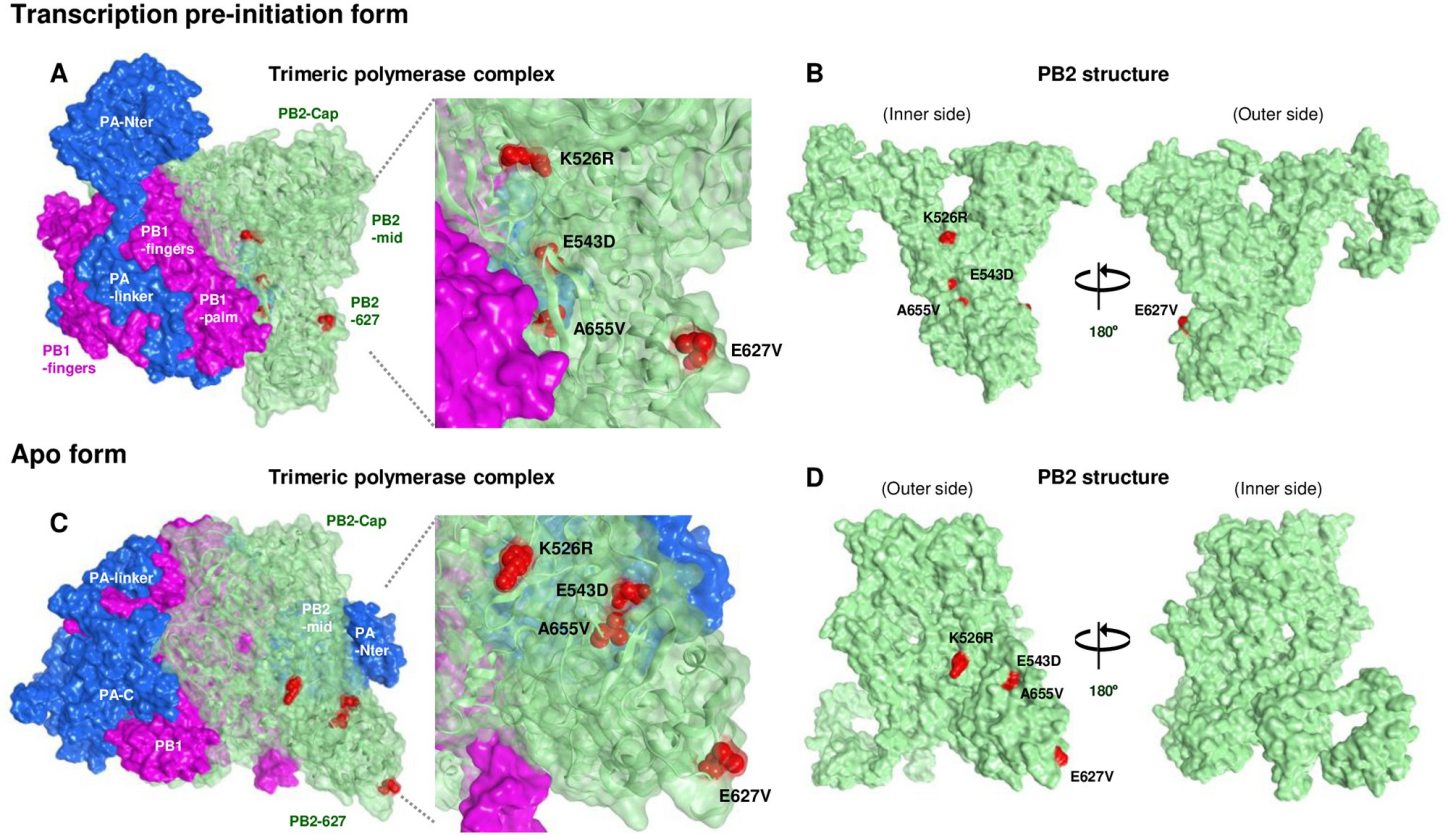
Location of PB2 mutations on the structure of the trimeric G1/1997 polymerase complex. (A and C) Transparent surface diagram of the G1/1997 polymerase complex composed of PB2 (green), PB1 (pink) and PA (blue) with E627V, D543E, A655V and K526R mutations (red). (A) The transcription pre-initiation form (PDB: 4WSB as the modeling template) and (C) the apo form (PDB: 5D98 as the modeling template) are shown. (B and D) Surface view of the PB2 structure in the transcription pre-initiation form (B) and the apo form (D). The left and right PB2 structures show the outer side and the inner side of the polymerase complex, as indicated, and differ by 180° in orientation.

The influenza virus polymerase complex is intrinsically flexible and can adopt multiple conformations depending on the presence or absence of specific RNA [25–27]. Our structural model of the heterotrimeric G1/1997 polymerase complex showed that PB2 mutations that increased polymerase activity and replication in human cells and mice (i.e., E627V, E543D, A655V and K526R) were exposed on the surface of the PB2-627 domain in at least one of the two conformations of the polymerase complex that we examined. In the transcription pre-initiation form (PDB: 4WSB as the modeling template), E543D, A655V and K526R were located on the inner side of the polymerase complex (Fig 7A and 7B). E543D was located on the PB2 surface, but A655V was less surface-exposed. The two sites were close to PB1, but not directly contacting it. K526R faced the product exit channel. In addition, E627V was fully exposed on the surface at outer side of the polymerase complex. In the rearrangement of the polymerase conformation from the transcription pre-initiation form to the apo form (PDB: 5D98 as the modeling template), all of the mutations became more fully exposed on the surface (Fig 7C and 7D) and located at the outer side of polymerase complex. These results suggested that the mutations identified here may affect interaction between the PB2-627 domain and host factor(s) that mediate species-pecific effects to promote polymerase activity and viral replication.

In the apo form of the polymerase complex, our model indicated that PB2 residues 543 and 655 were located close together (Fig 7C and 7D). This was in agreement with the results of our minigenome assays and infection studies showing that the two mutations needed to be coupled in the G1/1997 genetic background to produce an appropriate polymerase function. These results suggested that multiple mutations emerged coordinately to structurally optimize the polymerase complex for increased fitness in hosts. Although our structural study was based on a plausible model, future structural analyses based on crystallography, cryo-EM and better defined structural models are needed to investigate the detailed mechanism of polymerase mutations in different functional states, as described elsewhere [25].

## Discussion

The influenza virus H9N2 G1-like clade continues to evolve and expand its geographic host range in Central Asia and the Middle East. These areas include most predominantly Islamic nations, which have significant poultry population but minimal swine production. In contrast, East Asia, centered on China, has large swine production [28]. Regional clustering has led to the establishment of distinct influenza virus G1-like subclades (A-D), with subclade A and B strains most prevalent in the strains co-circulating in the Middle East [7]. Due to the increasing viral genetic diversity in a wide range of bird species, mainly poultry, it was inevitable that H9N2 G1 viruses would evolve to produce strains with distinct replication phenotypes. In particular, since the G1-A/B reassortant has been prevalent in domestic poultry in Egypt [13], with four cases of human infections, Egypt has been an epicenter of H9N2 evolution. This has produced novel viruses that have accumulated mutations and a large gene pool to enable reassortment with other influenza viruses. However, the biological properties of the H9N2 virus and its evolution in the Middle East have remained poorly understood.

Here we have elucidated the phylogeny of the G1-like clade in Central Asia and the Middle East and investigated its host range-associated mutations. Our results showed that the G1-like clade strains in these areas accumulated multiple PB2 mutations during their evolution in poultry. Four of the conserved mutations acted cooperatively in a G1-A/B reassortant that had an increased replication capacity in mammalian species. To our knowledge, this study presents the first comprehensive data analyzing the relationship between the phylogeny of the H9N2 virus in the Middle East and its host range expansion to mammals.

**Fig 8:**
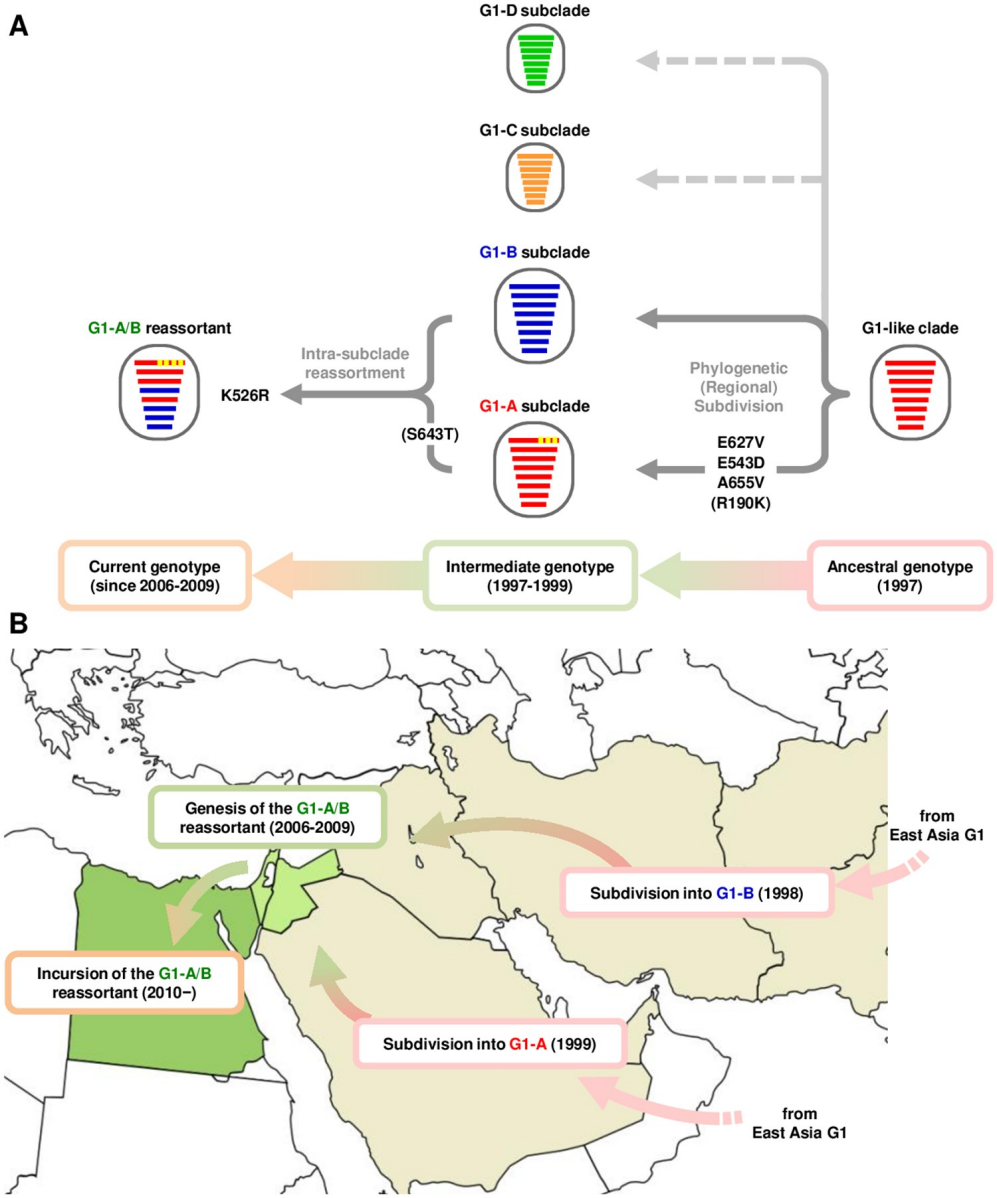
Evolutionary pathway of the G1-A/B reassortant in Central Asia and the Middle East. (A) Virus particles are shown as ovals with horizontal bars that represent the eight gene segments (from top to bottom: PB2, PB1, PA, HA, NP, NA, M and NS). The segments are colored according to the corresponding source viruses to indicate gene ancestry through reassortment. Yellow in gene segment 1 (PB2) indicates phylogeny-associated mutations that contribute to an expanded host range to humans. (B) Map of the Central Asia and the Middle East showing the phylogeny of G1-like subclades A and B and the G1-A/B reassortant in this study.

In this study, the PB2 E627V, E543D, A655V and K526R mutations acted cooperatively and produced increased replication of the G1-A/B reassortant in human cells and mice. However, the PB2-R190K and S643T mutations only had marginal effects. This was consistent with the hypothesis that the H9N2 virus PB2 gene was a key determinant in the host adaptation of AI viruses to mammals [29]. The G1-like clade accumulated the E627V, E543D, A655V and R190K mutations during phylogenetic branching from the ancestral G1-like clade to form the G1-A subclade in the Middle East (1997–1999) (summarized in Fig 8). The S643T mutation occurred during the time G1-A was prevalent in Israel and Jordan (1999–2006), and the K526R mutation occurred during the emergence of the G1-A/B reassortant in this area in about 2006. The resulting G1-A/B reassortant has retained all these mutations and has been prevalent in Egypt since 2010. This indicated that these phylogeny-associated PB2 mutations provided a selective advantage for adaptation of G1-like clade strains to poultry during its evolution in the field. Indeed, these mutations, singly and especially in combination, produced a moderate increase in G1/1997 polymerase activity and replication in avian cells, which may have led to the current prevalence of the G1-A/B reassortant in Egyptian poultry. H9N2 virus adaptation to poultry could have facilitated expansion of the viral host range, including infection of humans and other mammals [30]. The G1-like clade also has the ability to infect a wide range of host species, including wild birds, pigeons, pigs and humans [2], which may have generated more complicated phenotypes in the field. Taken together, these findings suggested that the G1-A/B reassortant evolved to produce strains with diverse phenotypes in the Middle East, which led to its expanded host range, including to humans.

Several of the mutations identified in this study have been previously described in other AI viruses. PB2-E627K is the best characterized human-adaptation mutation in AI viruses, particularly H5N1 viruses [31, 32], and is readily acquired by H9N2 viruses during passage in mice [33, 34]. PB2-E627V has been recently shown to increase H5N1 virus replication in human cells [23]. In addition, PB2-K526R is known to be linked to human adaptation [35] and has been found in human infections by Asian H5N1, H7N9 and H9N2 viruses. The K526R mutation has been occasionally found together with E627K and these two mutations have been shown to act cooperatively in mammalian adaptation in the H7N9 background [35]. All AI viruses in birds in Asia retain 627E and 526K. In contrast, Middle Eastern avian H9N2 viruses have commonly accumulated both E627V and K526R during evolution of the G1-A/B reassortant, thereby increasing viral adaptation in birds and mammalian species. Also, the H5N1 clade 2.2.1 virus that is unique to Egypt has generally accumulated E627K in the field [16, 36]. An important question is what has driven the distinct evolutionary dynamics of the H9N2 and H5N1 viruses between the Middle East and Asia. It is possible that an early compensatory or enabling mutation(s) (e.g., A655V) in the Middle Eastern G1 lineage allowed the accumulation of adaptive mutations distinct from those observed in other avian influenza virus lineages. Selective pressure in the Middle East may differ from that in Asia. However, it is difficult to identify specific determinant(s) because multiple uncertainties have contributed to AI epidemics, including changes in environmental, behavioral, and social factors and changes in the poultry trade [28]. Also, the S543F mutation, but not the E543D mutation, was recently suggested to be linked to human adaption of H7N9 viruses by a prospective approach to deep mutational scanning [37]. A655V has not been previously described as an adaptation mutation in AI viruses, including H5N1, H7N9 and H9N2 viruses.

The exact mechanism of the effects of the PB2 mutations identified in this study remains unknown. These mutations clustered on the surface of the PB2 627 domain, which is a solvent-exposed region of the polymerase complex in both its transcription pre-initiation form and apo form. This suggested the possibility that the mutations may affect the interactions between the PB2-627 domain and host factor(s), such as the ANP32A proteins that have been shown to be involved in host species specificity mediated by the influenza virus PB2-627 domain [25, 38–40]. It is also possible that the mutations may not make direct contact with host factor(s), but may affect PB2 flexibility to enable other viral domains to promote interactions with host factor(s). In addition, in this study, the higher mortality and severe weight loss of mice infected with these influenza virus adaptation mutations was in agreement with our *in vitro* studies showing higher polymerase activity and viral RNA synthesis and virus titers in the lungs and nasal turbinates. This indicated that increased replication fitness contributed to high influenza virus virulence in mice. However, several studies reported that influenza A virus polymerase can produce additional viral RNA products, often called aberrant RNAs, which trigger an innate immune response [41]. Thus, there remains the possibility that the more active mutant polymerases may produce more of these aberrant RNAs, which may be partially associated with the higher virulence of the adaptive mutants in this study in infected mice.

The H9N2 virus and the H5N1 clade 2.2.1 viruses have been prevalent in poultry in Egypt, which has provided the opportunity for reassortment between the two viruses. Fortunately, the emergence of H9N2/H5N1 reassortants has not been reported in Egypt thus far. However, we recently reported that Egyptian H9N2 and H5N1 viruses have a high genetic compatibility and a substantial potential to generate reassortants with greater fitness in mammalian species than the parental viruses [20]. The results of this study suggested that, if an H5N1 clade 2.2.1 virus successfully recruited the G1-A/B PB2 gene, such reassortants may pose an infection threat beyond the current outbreaks in the Middle East. The G1-A/B reassortant has an increased replication ability in human cells at 33°C and in the upper respiratory tract of mice. This implied that an exposure to fewer G1-A/B reassortant viruses may establish an infection in the respiratory tract of a mammalian species, greatly increasing the likelihood of a co-infection and reassortment with a seasonal human or swine influenza virus in the upper airway. The G1-A/B reassortant has an HA-Q226L mutation (H3 numbering) that increased viral binding affinity to human respiratory epithelia [42]. In view of this concern, it should be noted that seroepidemiological studies have indicated substantial exposure of humans and pigs to both H5N1 and H9N2 viruses in Egypt [43, 44]. A high level of genetic compatibility between H9N2 and seasonal H1N1 2009 viruses also has been reported [45, 46]. Taken together, these findings suggested that the Middle East, with an epicenter in Egypt, is now a hot spot of H9N2 and H5N1 virus evolution, which may generate novel viruses presenting an increased public health risk.

In conclusion, this study showed that the influenza virus G1-A/B reassortant, that has been prevalent in Egypt, has diversified phylogenetically in the Middle East during its natural evolution and generated a distinct phenotype with an expanded host range to mammals, including humans. Our results highlighted the need for studies of H9N2 viruses to trace their evolutionary dynamics in this area and closely monitor possible genetic changes in these viruses. In addition, implementation of effective control measures may be needed to reduce the risk of a future pandemic.

## Materials and methods

### Ethics statement

Ten-day-old embryonated chicken eggs were purchased from Shimizu Laboratory Supplies, Japan. All animal experiments were conducted in compliance with Japanese legislation (Act on Welfare and Management of Animals, 1973, revised in 2012) and guidelines under the jurisdiction of the Ministry of Education, Culture, Sports, Science and Technology in Japan. Animal care, housing, feeding, sampling, observation, and environmental enrichment were approved by the Animal Experiment Committee of the Kyoto Prefectural University of Medicine (Approval number M29-554 and M30-60).

### Biosecurity and biosafety

All experiments with live H9N2 viruses were performed at enhanced Biosafety Level 3+ (BSL3+) in Kyoto Prefectural University of Medicine. All studies with recombinant DNA were conducted under the relevant laws in Japan and approved by the Biological Safety Committee of Kyoto Prefectural University of Medicine (Approval number 30–104) after risk assessments were conducted by the Living Modified Organisms Committee of Kyoto Prefectural University of Medicine and, when required, by the Ministry of Education, Culture, Sports, Science and Technology of Japan.

### Cells and viruses

Human bronchial epithelial (Calu-3) cells and chicken fibroblast (DF-1) cells were obtained from the American Type Culture Collection, and MDCK cells and 293T cells were obtained from the RIKEN BioResource Center Cell Bank. The cells were maintained in Dulbecco’s Modified Eagle’s Medium with 10% FCS. Influenza virus A/chicken/Egypt/CL42/2013 (EG/2013) (H9N2) is a representative strain of the G1-A/B reassortant that has been prevalent in Egypt and has been described previously [20]. All recombinant H9N2 viruses were propagated once in 10-day-old embryonated eggs. The sequences of the EG/2013 gene segments have been deposited in the GenBank database (accession nos. LC379963-LC379970).

### Generation of viruses by reverse genetics

As described previously [47, 48], recombinant viruses were generated with a plasmid-based reverse genetics system in the EG/2013 (H9N2) genetic background. Mutations were introduced into the plasmids using PCR-based site-directed mutagenesis. All propagated viruses were completely sequenced to ensure the absence of unwanted mutations.

### Minigenome assays

293T and DF-1 cells were transfected with pCAGGS plasmids encoding the PB2, PB1, PA or NP genes of EG/2013 (H9N2), G1/1997 (H9N2), EG/2007 (H5N1) and Sh/2013 (H7N9) and a human or chicken polymerase-I driven plasmid expressing firefly luciferase as described previously [16, 47]. A plasmid expressing *Renilla* luciferase was also co-transfected to monitor transfection efficiencies. The transfected cells were incubated at 33°C or 37°C and lysed at 24 h post-transfection. The firefly luciferase activities were normalized relative to *Renilla* luciferase activity.

### Phylogenetic analyses

The sequences of the PB2 and HA gene segments from reference H9N2 strains were obtained from the GISAID database (http://www.gisaid.org). Phylogenetic analysis was performed using MEGA6 software for the neighbor-joining method, with the nucleotide sequences of representative strains of the H9N2 G1-like clade from Central Asia and the Middle East, including prototype G1/1997 and EG/2013 viruses, and of A/duck/Hong Kong/Y280/1997 as the prototype of the H9N2 Y280-like clade virus. Although a few human and swine H9N2 virus infections have been reported in Central Asia and/or the Middle East, the human- and swine-derived H9N2 sequences were not included in these analyses, because they have not yet been deposited in a pubic database. Confidence levels for phylogenetic trees were calculated by performing 1,000 bootstrap replicates.

### Immunoprecipitation of reconstituted polymerase complexes

For PB2-flag immunoprecipitation, 293T cells in 10 cm dishes were transfected with pCAGGS plasmids encoding PB2-flag, PB1 and PA genes of EG/2013 (H9N2) and G1/1997 (H9N2). Cells were harvested at 48 h post-transfection in cold PBS and lysed in 700 μl Tris lysis buffer (50 mM Tris-HCL [pH 8.8], 1 mM DTT, 0.5% Igepal, 25% glycerol, 200 mM NaCl) with Complete Mini EDTA-free protease inhibitor cocktail (Promega) at 4°C for 40 min. The lysates were cleared by centrifugation at 800 g for 5 min. Anti-Flag M2 Agarose Affinity Gel (Sigma-Aldrich) was added to the cleared lysates and they were mixed at 4°C for 2 h. The mixtures were washed 3 times with the Tris lysis buffer and purified PB2-flag, and PB1 and PA were analyzed by western blotting.

### Western blotting

For expression analyses, 293T cells were transfected with equal amounts of plasmids encoding the non-tagged or flag-tagged PB2 gene of EG/2007 (H5N1), Sh/2013 (H7N9) or EG/2013 (H9N2). At 16 h post-transfection, the cells were harvested with sample buffer. For the PB2-flag immunoprecipitation assays described above, final immunoprecipitates were boiled in sample buffer and analyzed by western blotting. The samples were separated by SDS-PAGE and transferred onto polyvinylidene difluoride membranes (Millipore). Immunoblot analysis was performed using anti-influenza PB2, PB1 and PA antibody (GeneTex), anti-flag antibody (Sigma-Aldrich) and HRP-conjugated secondary antibody. The bands were visualized with Amersham ECL Select Western Blotting Detection Reagent and the Amersham Imager 600 (GE Healthcare). The band intensities were quantified by Amersham Imager 600 Analysis Software (GE Healthcare).

### RNP reconstitution and strand-specific real-time RT-PCR

293T cells and DF-1 cells were transfected with pCAGGS plasmids encoding the wild-type or mutant PB2, PB1, PA or NP genes of EG/2013 and G1/1997 and a human or chicken polymerase-I driven plasmid encoding a full-length NA vRNA or cRNA template to reconstitute the NA RNP. At 24 h post-transfection, viral RNAs were extracted from the cells with an RNeasy Mini Kit (Qiagen) and treated with RNase-free DNase Set (Qiagen) to eliminate any residual transfected plasmid DNA. cDNAs derived from NA vRNA, cRNA and mRNA were reverse transcribed with the tagged primers listed in S1 Table and the hot-start modification of the SuperScript III first-strand synthesis system (Invitrogen) using saturated trehalose [49]. The same tags, as described elsewhere [49], were used and the primers were designed to target both EG/2013 and G1/1997 NA. Quantitative real-time PCR (qPCR) was performed using the PowerUp SYBR Green Master Mix (ThermoFisher) with the strand-specific primers listed in S1 Table. The qPCR cycle conditions were 50°C for 2 min and 95°C for 2 min, followed by 40 cycles of 95°C for 15 sec and 60°C for 1 min. Serial 10-fold dilutions (10^9^, 10^8^, 10^7^, 10^6^, 10^5^, 10^4^, 10^3^ and 10^2^ copies) of *in vitro* transcribed viral RNA standards were used to generate a standard curve. The specificity of strand-specific qRT-PCR was tested using an NA gene that was amplified by PCR using a human polymerase-I driven plasmid encoding a full-length EG/2013 NA vRNA template. The primers, listed in S2 Table, were designed to include T7 promoter sequences to produce template vRNA, cRNA and mRNA. PCR products were purified by a PCR Purification Kit (Qiagen). Template vRNA, cRNA and mRNA were transcribed *in vitro* using the RiboMax Large-Scale RNA Production System-T7 (Promega). Reverse transcription was performed with 10^9^ copies of each template RNA (vRNA, cNRA and mRNA) using the tagged primers described above. The cDNA products were quantified by qPCR reactions using the strand-specific primer sets described above.

### Viral infection of cultured cells

DF-1 and Calu-3 cells (90% confluent in collagen-coated 24-well plates) were infected in triplicate with the indicated viruses at an MOI of 0.005 and 0.001, respectively. After 1 h at 37°C, the cells were washed with PBS and maintained in DMEM-F12 containing 0.2% BSA and 2 μg acetylated trypsin/ml (Sigma-Aldrich) at 33°C or 37°C. The supernatants were collected at the indicated times post-infection and viral titers were determined by assays of focus-forming units (FFU) as described previously [47].

### Viral infection of mice

Four to five-week-old female BALB/c mice (Japan SLC), under mixed anesthesia (medetomidine-butorphanol-midazolam), were inoculated intranasally with 50 μl samples of 10-fold serial dilutions of viruses in PBS. The body weight and survival of each mouse was monitored daily for 14 d. Mice that lost more than 30% of their original weight within a few days were humanely euthanized. Lungs and nasal turbinates of mice infected with 10^4^ FFU virus were collected 3 dpi and virus titers were assayed by FFU assays. For histopathological analysis, lungs of mice infected with 10^4^ FFU were collected at 3 dpi, fixed in 4% buffered paraformaldehyde, embedded in paraffin, cut into 5 μm sections, stained with hematoxylin and eosin, and examined by light microscopy. Immunohistochemical straining of viral antigen was performed as described previously [16], using anti-NP antibody and a Mouse on Mouse Peroxidase Kit (Vector Laboratories) with diaminobenzidine as the chromogen and hematoxylin as the counterstain.

### Homology modeling of the polymerase complex

The transcription pre-initiation form and the apo form of the G1/1997 trimeric polymerase complex of the indicated G1/1997 mutants were modeled using published crystal structures of the influenza virus polymerase complexes (PDB: 4WSB and 5D98, respectively). MOE was used for software and most of the modeling conditions were described previously [16, 47, 50]. As a point of change from the previous studies, the number of side chain samples was set to 5.

### Statistical analysis

Statistical analysis was carried out using GraphPad Prism Version 6 software (GraphPad Software Inc.). Statistically significant differences of virus pairs were determined by ANOVA with Tukey’s multiple comparison test.

## Supporting information

S1 Fig Quantification of PB2-flag expression in 293T cells. The cells were transfected with plasmids expressing the indicated flag-tagged PB2, and cell lysates were analyzed by western blotting using anti-flag antibody. Representative images are shown. Each data point is the mean ± SD of five independent experiments. NS indicates no statistically significant difference.
(TIF)

S2 Fig Phylogenetic tree of HA genes of viruses in the H9N2 G1-like subclade isolated in the Middle East and Central Asia, including the G1-A/B reassortant. The phylogenetic tree was reconstructed from nucleotide sequences of the HA genes of the Middle Eastern and Central Asian reference strains in the GISAID database, the G1-like clade prototype strain G1/1997, the G1-A/B reassortant strain EG/2013, and the Y280-like clade prototype strain A/duck/Hong Kong/Y280/1997. CK, DK, QL and TK in virus strain names denote chicken, duck, quail and turkey hosts. Virus particles are shown as ovals with horizontal bars representing the eight gene segments (from top to bottom: PB2, PB1, PA, HA, NP, NA, M and NS). Gene segments in the descendent viruses are colored according to their corresponding source viruses to illustrate gene ancestry through reassortment. Yellow in segment 1 (PB2) indicates phylogeny-associated mutations that contribute to expansion of the viral host range to humans.
(TIF)

S3 Fig Effects of single reverse mutations on EG/2013 polymerase activity in human cells at 37°C. Polymerase activity of EG/2013 and the PB2 revertants carrying single mutations was measured in human 293T cells at 37°C. The viruses were either not carrying PB2-V627E (A) or were carrying PB2-V627E (B). The data are expressed relative to the results for EG/2013-627V (wt) (A) and EG/2013-V627E (B). Colors of the vertical bars indicate the four mutation categories based on the time periods when the mutations were identified, as shown in Fig 2A.
(TIF)

S4 Fig Expression of the PB2 mutants in this study in human cells. Human 293T cells were transfected with PB2 expression plasmids carrying the indicated mutations and either not carrying V627E (A and B) or carrying V627E (C and D). At 16 h post-transfection, the cells were harvested and analyzed by western blotting using anti-PB2 antibody. Representative images are shown. (A and C). After quantification of the band intensities, the amount of expression of each PB2 construct was calculated relative to that for EG/2013-627V (wt). Each data point is the mean ± SD of five independent experiments. (B and D) Representative results of western blotting for EG/2013-627V (wt), the PB2 mutants and the reference EG/2013-G1/PB2. NS indicates no statistically significant difference.
(TIF)

S5 Fig Verification of strand specificity of the primers for viral NA vRNA, cRNA and mRNA. The strand specificity of the primers was verified using EG/2013 NA vRNA, cRNA and mRNA templates prepared by *in vitro* transcription as described in Materials and Methods. The specificity of primers for EG/2013 vRNA, cRNA and mRNA is shown with respect to the percent of the corresponding RNA template. Each data point is the mean ± SD of three independent experiments. These data indicated that the qRT-PCR primers were highly specific for distinguishing viral vRNA, cRNA and mRNA, as previously reported [49].
(TIF)

S6 Fig Cooperative effects of the E543D and A655V mutations on G1 replication kinetics in avian and human cells. Avian DF-1 cells (A) and human Calu-3 cells (B and C) were infected with G1(3P+NP) virus and PB2 mutants carrying E543D alone or in combination with the indicated mutations at an MOI of 0.005 and 0.001, respectively, and incubated at 37°C (A and B) or 33°C (C). Virus titers at the indicated times post-infection were determined by FFU assays. Each data point is the mean ± SD of three independent experiments. The data indicated that the E543E/A655V double mutation, but not the E543 single mutation, produced efficient G1 replication in avian and human cells, in agreement with the minigenome assay data in Fig 3A.
(TIF)

S1 Table Primers for strand-specific real-time RT-PCR using tagged primers for quantification of NA vRNA, cRNA and mRNA. (PDF)

S2 Table Primers for PCR to produce the templates for in vitro transcription of viral RNA reference standards for NA vRNA, cRNA and mRNA. (PDF)
