# Supplementary figures and images for: PB2 mutations arising during H9N2 influenza evolution in the Middle East confer enhanced replication and growth in mammals
Source: PLoS Pathog. 2019 Jul 2;15(7):e1007919. doi: 10.1371/journal.ppat.1007919 (PMC6629154; doi:10.1371/journal.ppat.1007919)

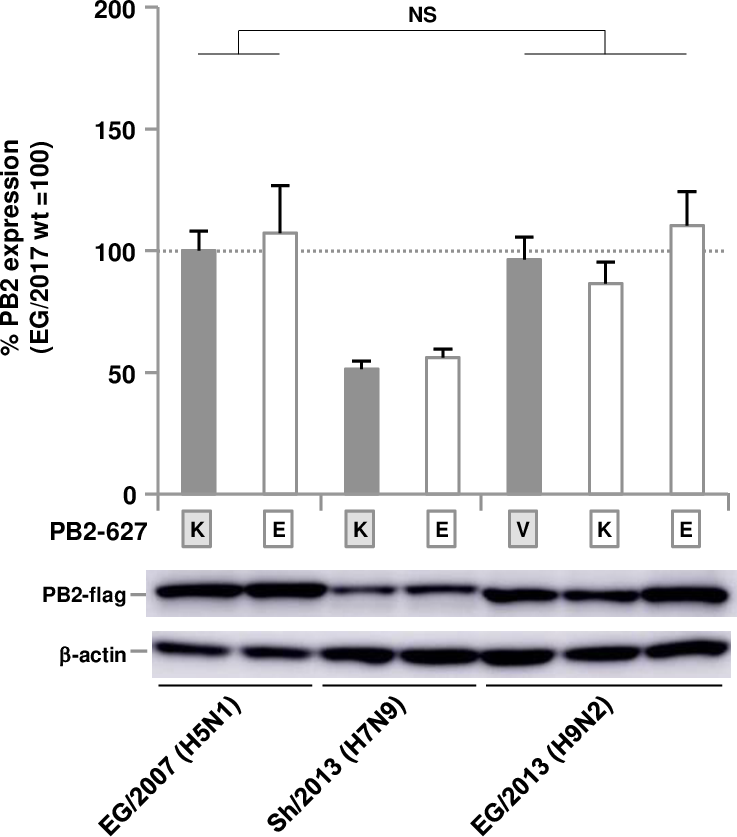

Supplement: S1 Fig — The cells were transfected with plasmids expressing the indicated flag-tagged PB2, and cell lysates were analyzed by western blotting using anti-flag antibody. Representative images are shown. Each data point is the mean ± SD of five independent experiments. NS indicates no statistically significant difference. (TIF) [file ppat.1007919.s001.tif]

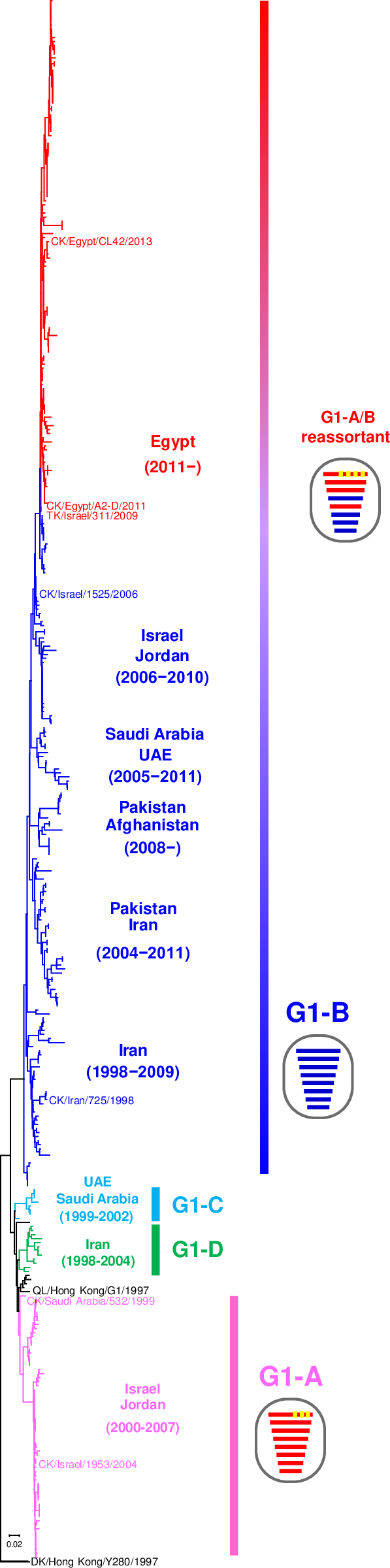

Supplement: S2 Fig — The phylogenetic tree was reconstructed from nucleotide sequences of the HA genes of the Middle Eastern and Central Asian reference strains in the GISAID database, the G1-like clade prototype strain G1/1997, the G1-A/B reassortant strain EG/2013, and the Y280-like clade prototype strain A/duck/Hong Kong/Y280/1997. CK, DK, QL and TK in virus strain names denote chicken, duck, quail and turkey hosts. Virus particles are shown as ovals with horizontal bars representing the eight gene segments (from top to bottom: PB2, PB1, PA, HA, NP, NA, M and NS). Gene segments in the descendent viruses are colored according to their corresponding source viruses to illustrate gene ancestry through reassortment. Yellow in segment 1 (PB2) indicates phylogeny-associated mutations that contribute to expansion of the viral host range to humans. (TIF) [file ppat.1007919.s002.tif]

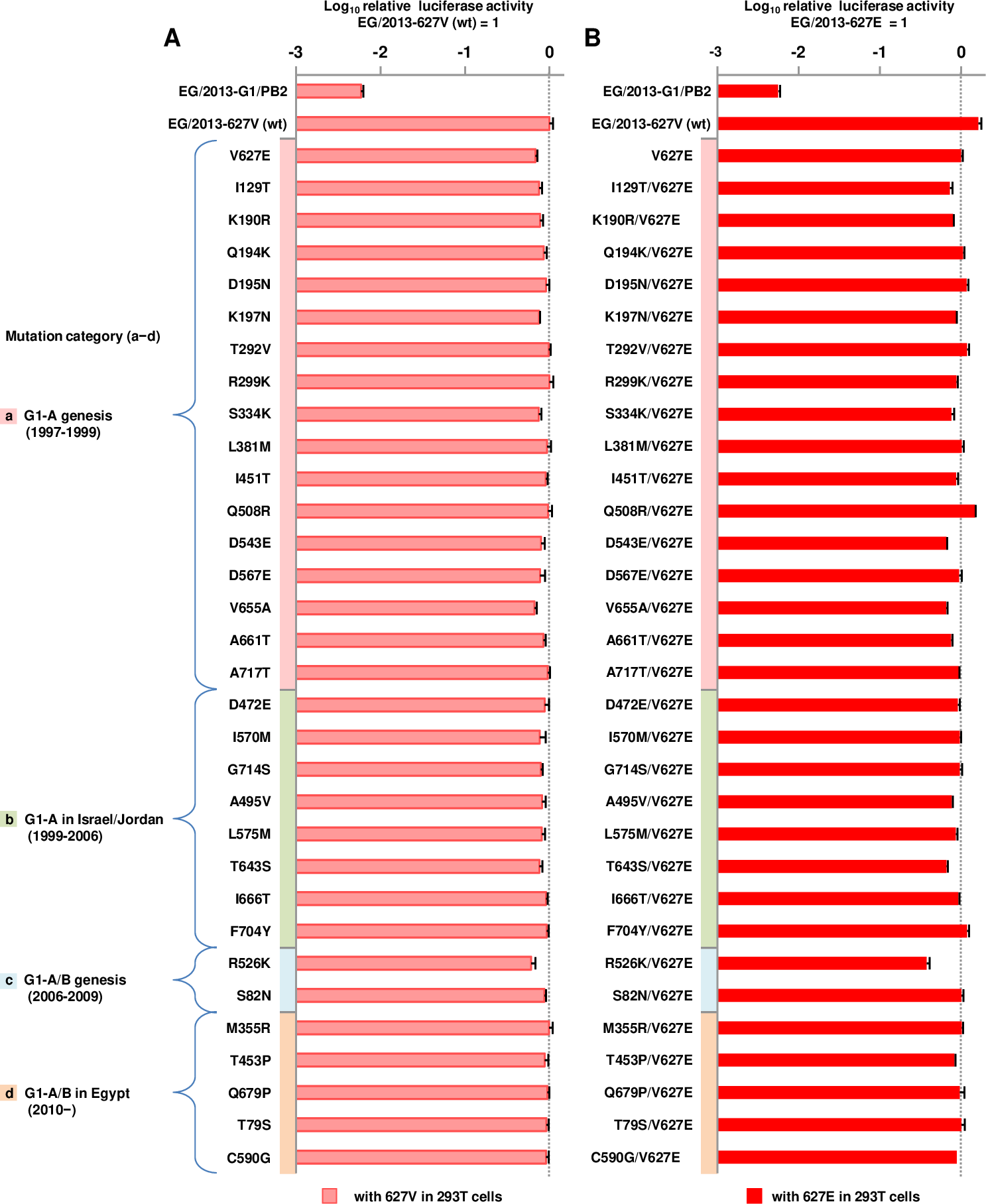

Supplement: S3 Fig — Polymerase activity of EG/2013 and the PB2 revertants carrying single mutations was measured in human 293T cells at 37°C. The viruses were either not carrying PB2-V627E (A) or were carrying PB2-V627E (B). The data are expressed relative to the results for EG/2013-627V (wt) (A) and EG/2013-V627E (B). Colors of the vertical bars indicate the four mutation categories based on the time periods when the mutations were identified, as shown in Fig 2A. (TIF) [file ppat.1007919.s003.tif]

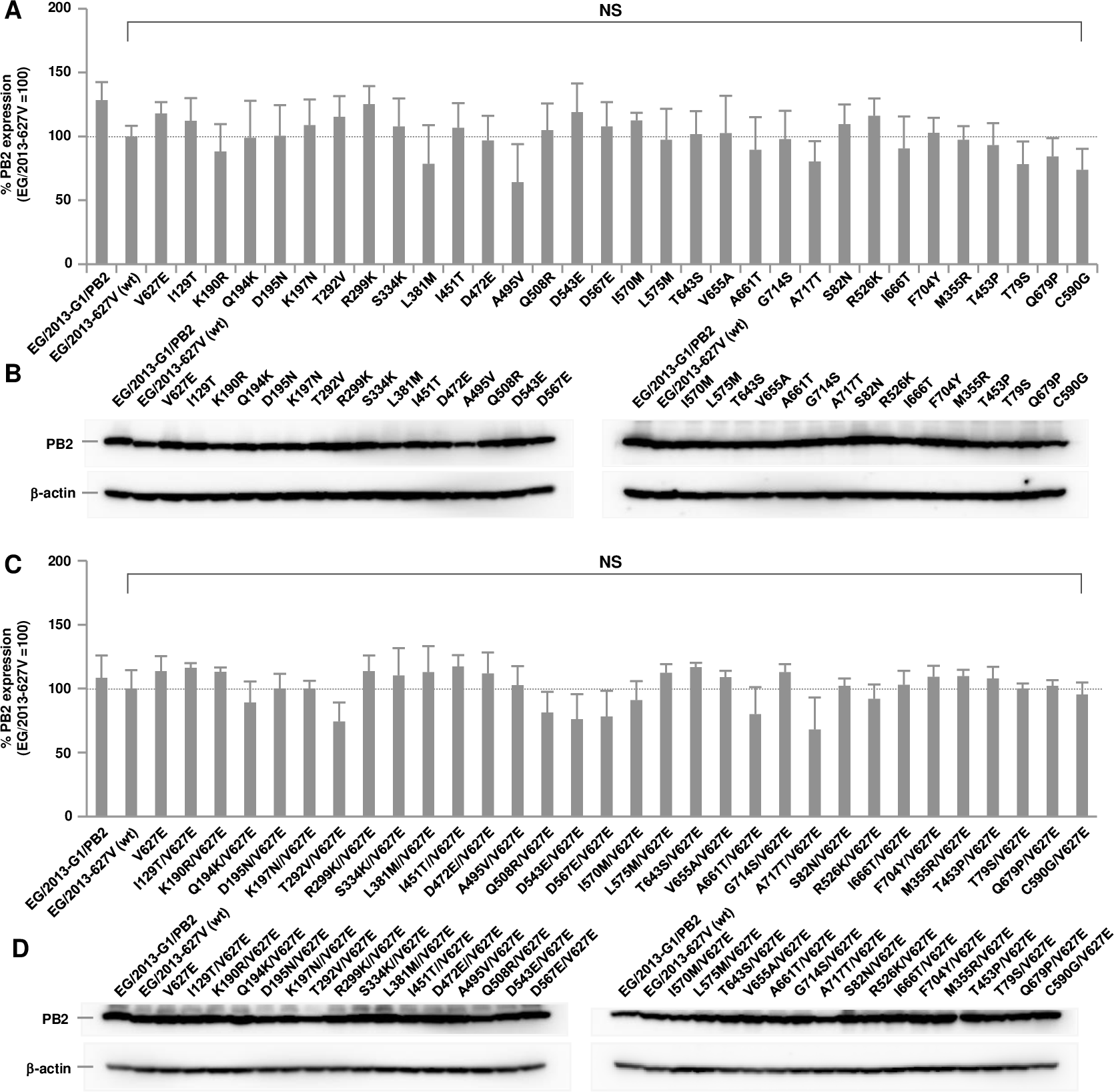

Supplement: S4 Fig — Human 293T cells were transfected with PB2 expression plasmids carrying the indicated mutations and either not carrying V627E (A and B) or carrying V627E (C and D). At 16 h post-transfection, the cells were harvested and analyzed by western blotting using anti-PB2 antibody. Representative images are shown. (A and C). After quantification of the band intensities, the amount of expression of each PB2 construct was calculated relative to that for EG/2013-627V (wt). Each data point is the mean ± SD of five independent experiments. (B and D) Representative results of western blotting for EG/2013-627V (wt), the PB2 mutants and the reference EG/2013-G1/PB2. NS indicates no statistically significant difference. (TIF) [file ppat.1007919.s004.tif]

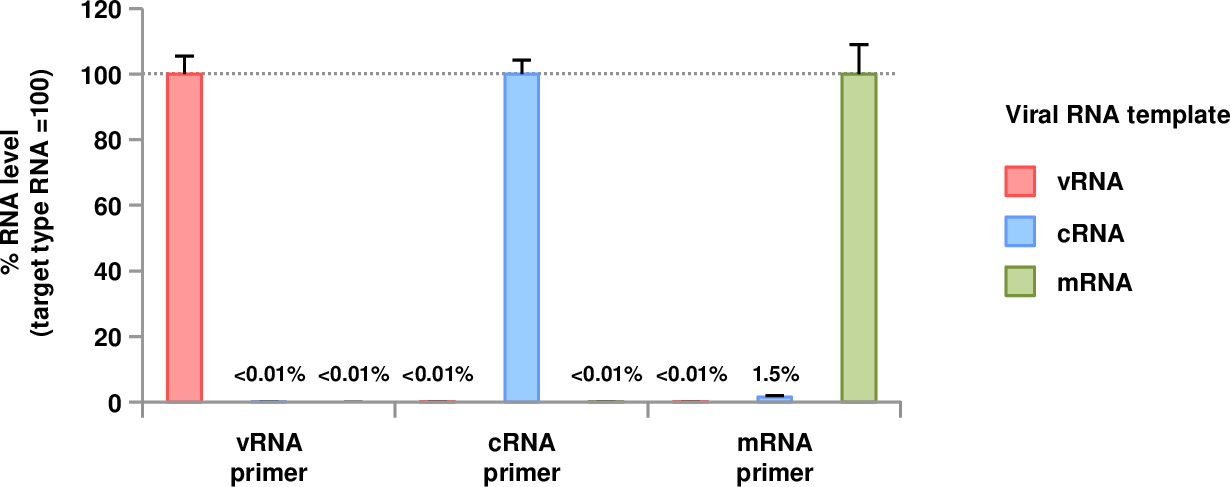

Supplement: S5 Fig — The strand specificity of the primers was verified using EG/2013 NA vRNA, cRNA and mRNA templates prepared by in vitro transcription as described in Materials and Methods. The specificity of primers for EG/2013 vRNA, cRNA and mRNA is shown with respect to the percent of the corresponding RNA template. Each data point is the mean ± SD of three independent experiments. These data indicated that the qRT-PCR primers were highly specific for distinguishing viral vRNA, cRNA and mRNA, as previously reported [49]. (TIF) [file ppat.1007919.s005.tif]

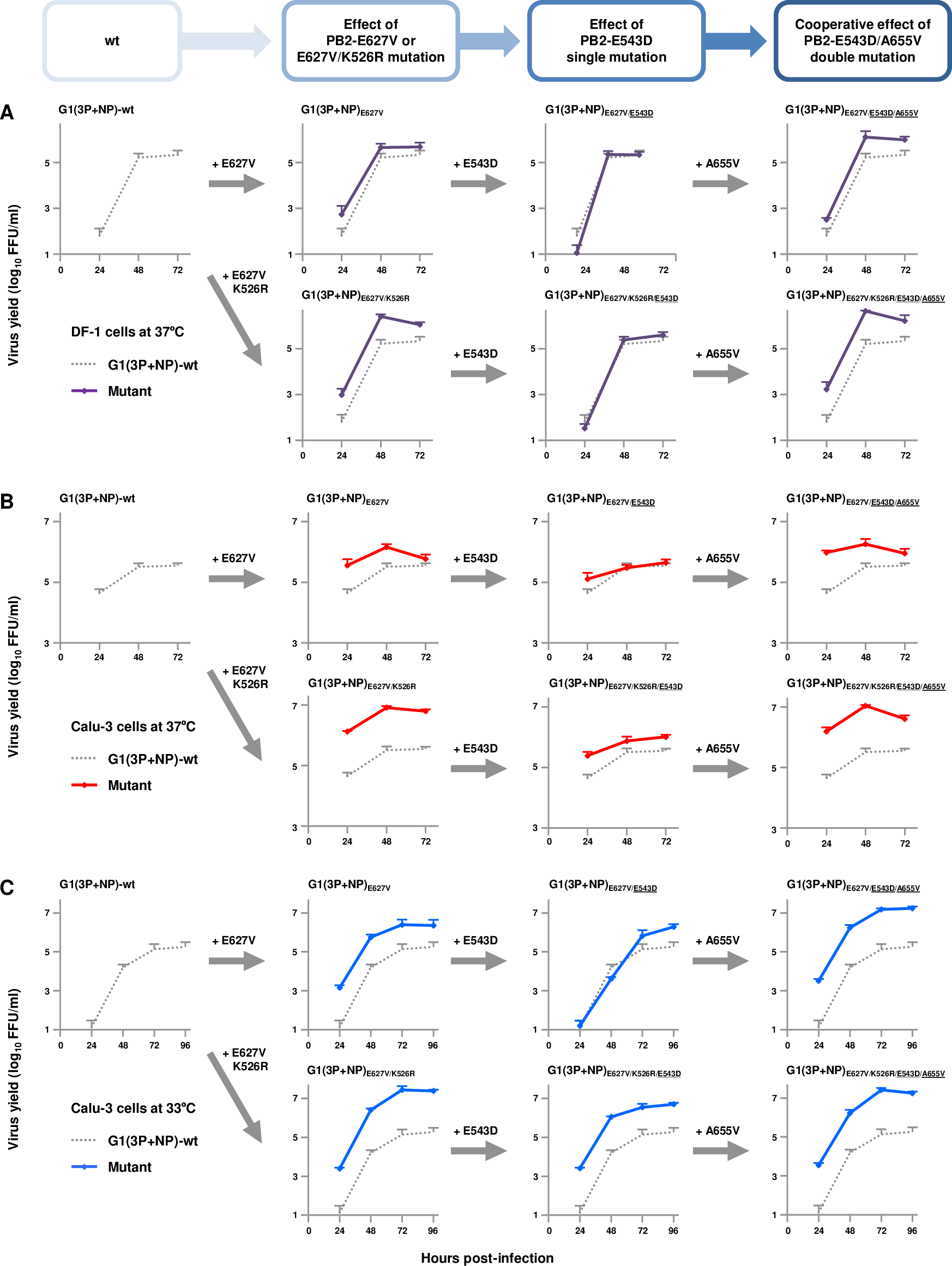

Supplement: S6 Fig — Avian DF-1 cells (A) and human Calu-3 cells (B and C) were infected with G1(3P+NP) virus and PB2 mutants carrying E543D alone or in combination with the indicated mutations at an MOI of 0.005 and 0.001, respectively, and incubated at 37°C (A and B) or 33°C (C). Virus titers at the indicated times post-infection were determined by FFU assays. Each data point is the mean ± SD of three independent experiments. The data indicated that the E543E/A655V double mutation, but not the E543 single mutation, produced efficient G1 replication in avian and human cells, in agreement with the minigenome assay data in Fig 3A. (TIF) [file ppat.1007919.s006.tif]
