# Supplementary material for: PB2 mutations arising during H9N2 influenza evolution in the Middle East confer enhanced replication and growth in mammals
Source: PLoS Pathog. 2019 Jul 2;15(7):e1007919. doi: 10.1371/journal.ppat.1007919 (PMC6629154; doi:10.1371/journal.ppat.1007919)
Supplement: S1 Table — (PDF) [file ppat.1007919.s007.pdf]

S1 Table. Primers for strand-specific real-time RT-PCR using tagged primers for quantification of NA vRNA, cRNA and mRNA.

Target	Purpose	Primer name	Sequences (5' to 3')
vRNA	Reverse transcription	vRNAtag_H9N2 G1seg6_730F	GGCCGTCATGGTGGCGAATTGCAGTAGTAATGACTGATGGAAG
	Real-time RT-PCR	vRNAtag	GGCCGTCATGGTGGCGAAT
		H9N2 G1seg6_844R	ATCTAACTTCTGGGTACCTAGGGT
cRNA	Reverse transcription	cRNAtag_H9N2 G1seg6_1466R	GCTAGCTTCAGCTAGGCATCAGTAGAAACAAGGAGTTTTTTCTAA
	Real-time RT-PCR	cRNAtag	GCTAGCTTCAGCTAGGCATC
		H9N2 G1seg6_1365F	CTTCAGGTACATATGGAACAGGCTCA
mRNA	Reverse transcription	mRNAtag_H9N2 G1seg6_dTR	CCAGATCGTTCGAGTCGTTTTTTTTTTTTTTTTTCTAAAATTGCG
	Real-time RT-PCR	mRNAtag	CCAGATCGTTCGAGTCGT
		H9N2 G1seg6_1365F	CTTCAGGTACATATGGAACAGGCTCA
