# Supplementary material for: PB2 mutations arising during H9N2 influenza evolution in the Middle East confer enhanced replication and growth in mammals
Source: PLoS Pathog. 2019 Jul 2;15(7):e1007919. doi: 10.1371/journal.ppat.1007919 (PMC6629154; doi:10.1371/journal.ppat.1007919)
Supplement: S2 Table — (PDF) [file ppat.1007919.s008.pdf]

S2 Table. Primers for PCR to produce templates for *in vitro* transcription of viral RNA reference standards for NA vRNA, cRNA and mRNA.

Target	Primer name	Sequences (5' to 3')
vRNA	H9N2 G1seg6_vRNA_1F	AGCAAAAGCAGGAGTGAAAATGAA
	T7_H9N2 G1seg6_vRNA_1466R	GGATCCTAATACGACTCACTATAGGGAGTAGAAACAAGGAGTTTTTTCTA
cRNA	H9N2 G1seg6_cRNA_1466R	AGTAGAAACAAGGAGTTTTTTCTAA
	T7_H9N2 G1seg6_cmRNA_1F	GGATCCTAATACGACTCACTATAGGGAGCAAAAGCAGGAGTGAAAATGAA
mRNA	H9N2 G1seg6_mRNA_dTR	TTTTTTTTTTTTTTTTTCTAAAATTG
	T7_H9N2 G1seg6_cmRNA_1F	GGATCCTAATACGACTCACTATAGGGAGCAAAAGCAGGAGTGAAAATGAA
